# Supplementary material for: Zebrafish Retinal Ganglion Cells Asymmetrically Encode Spectral and Temporal Information across Visual Space
Source: Curr Biol. 2020 Aug 3;30(15):2927–2942.e7. doi: 10.1016/j.cub.2020.05.055 (PMC7416113; doi:10.1016/j.cub.2020.05.055)
Supplement: Document S1. Figures S1–S6 [file mmc1.pdf]

Current Biology, Volume *30*

Supplemental Information

Zebrafish Retinal Ganglion Cells Asymmetrically Encode Spectral and Temporal Information across Visual Space

Mingyi Zhou, John Bear, Paul A. Roberts, Filip K. Janiak, Julie Semmelhack, Takeshi Yoshimatsu, and Tom Baden

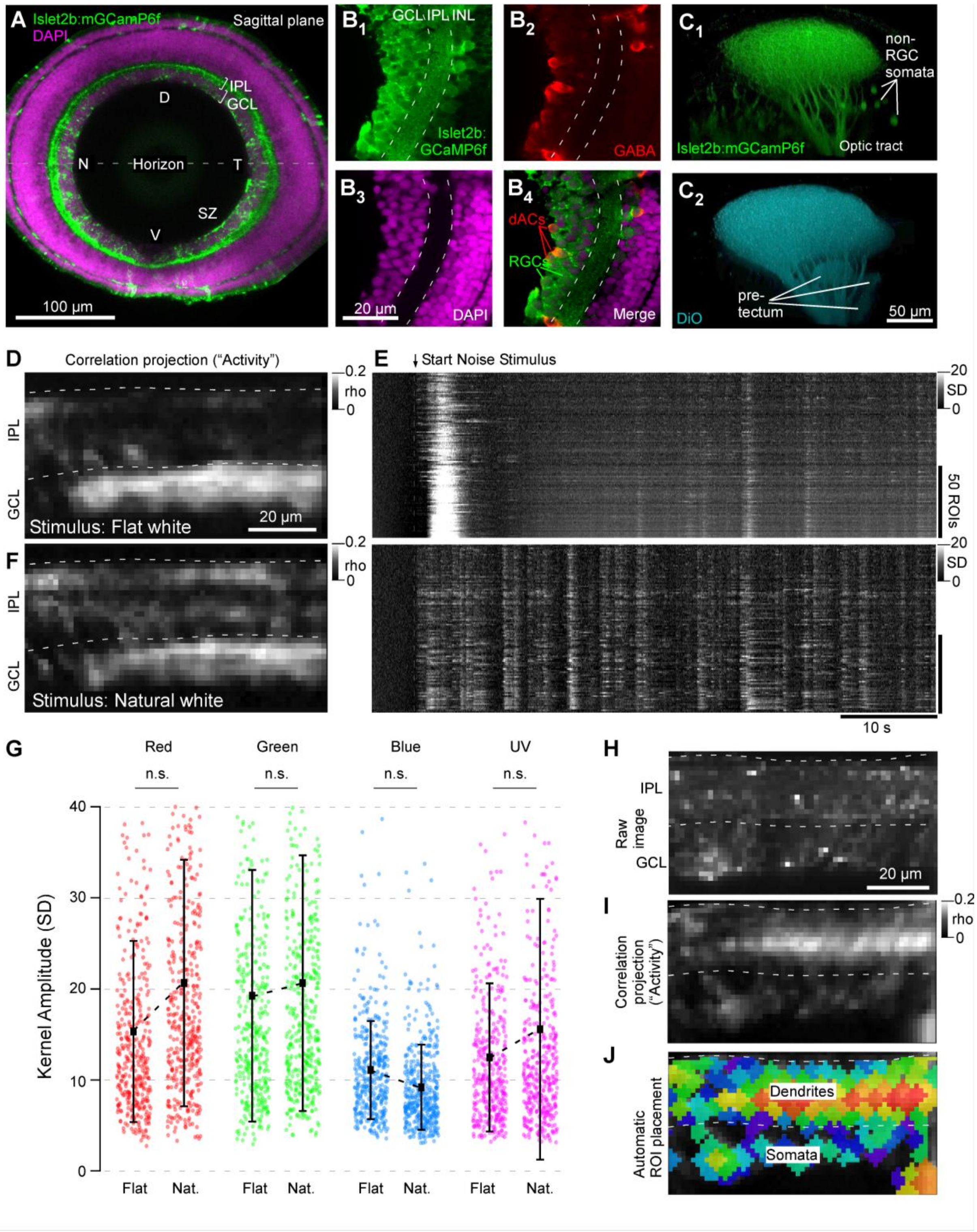

Figure S1. Islet2b expression, stimulation with "natural" white light and ROI placement. Related to Figure 1A. 7 dpf larva whole-eye sagittal plane confocal image of Islet2b:GCaMP6f expression (green) on the background of a DAPI stain, labelling all somata (magenta). D, dorsal; T, temporal; V, ventral; N, Nasal; SZ, Strike zone; INL Inner nuclear layer; GCL, Ganglion cell layer. **B$_{1\text{-}4}$,** Example higher magnification as in (A) from a second animal, with additional immunolabelling for GABA (red) to reveal GABAergic dACs and AC. Note the subset of somata showing both GABA labelling and mGCaMP6f expression (B$_4$). Note also the near doubling of GCL thickness across the region from the ventral retina (bottom) leading into the SZ (top). **C,** confocal projections of mGCaMP6f signal in the brain (C$_1$) and counter labelling by DiO injection in the eye (C$_2$). Though generally similar, C$_1$ shows expression in small numbers of brain-somata, while C$_2$ shows stronger labelling in pre-tectal

axonal arborisation fields. **D-F,** comparison of light-evoked activity in the same scan region in the SZ during stimulation with spectrally-flat white-noise (D, E top) and identical sequence ‘natural spectrum’ white noise (i.e. with green, blue and UV attenuated relative to red, cf. Figure 1B) (F, E bottom). Panels D,F show the correlation projection for the entire scan, while E top and bottom shows heatmaps of all extracted ROIs from each scan, respectively. Note the strong initial response when starting the noise-stimulus in the flat-white condition (E, top), followed by an extended period of response suppression. In contrast, ROIs during the natural-white condition responded briskly to the noise sequence without showing strong adaptation (E, bottom). Similarly, a more diverse set of scan-regions strongly responded in the natural white condition (F) compared to flat-white (D). **G**, The mean of the distributions of resultant kernel amplitudes across n=6 such scans from an identical number of animals (n=388 and 428 ROIs for the flat and natural-white condition, respectively) were indistinguishable (Wilcoxon Rank Sum Test, 2 tailed). Based on these results, we decided to use natural-white noise stimulation throughout this study. **H-J**, example scan demonstrating typical automated ROI placement. The scan was manually segmented into IPL (H, top) and GCL (H, bottom). In parallel, we computed mean correlation over time between all pairs of neighbouring pixels for the entire scan (I), and the resultant correlation-projection image was in turn used to seed and flood-fill ROIs (J). For further details and a discussion about the rationale of this approach, see [41].

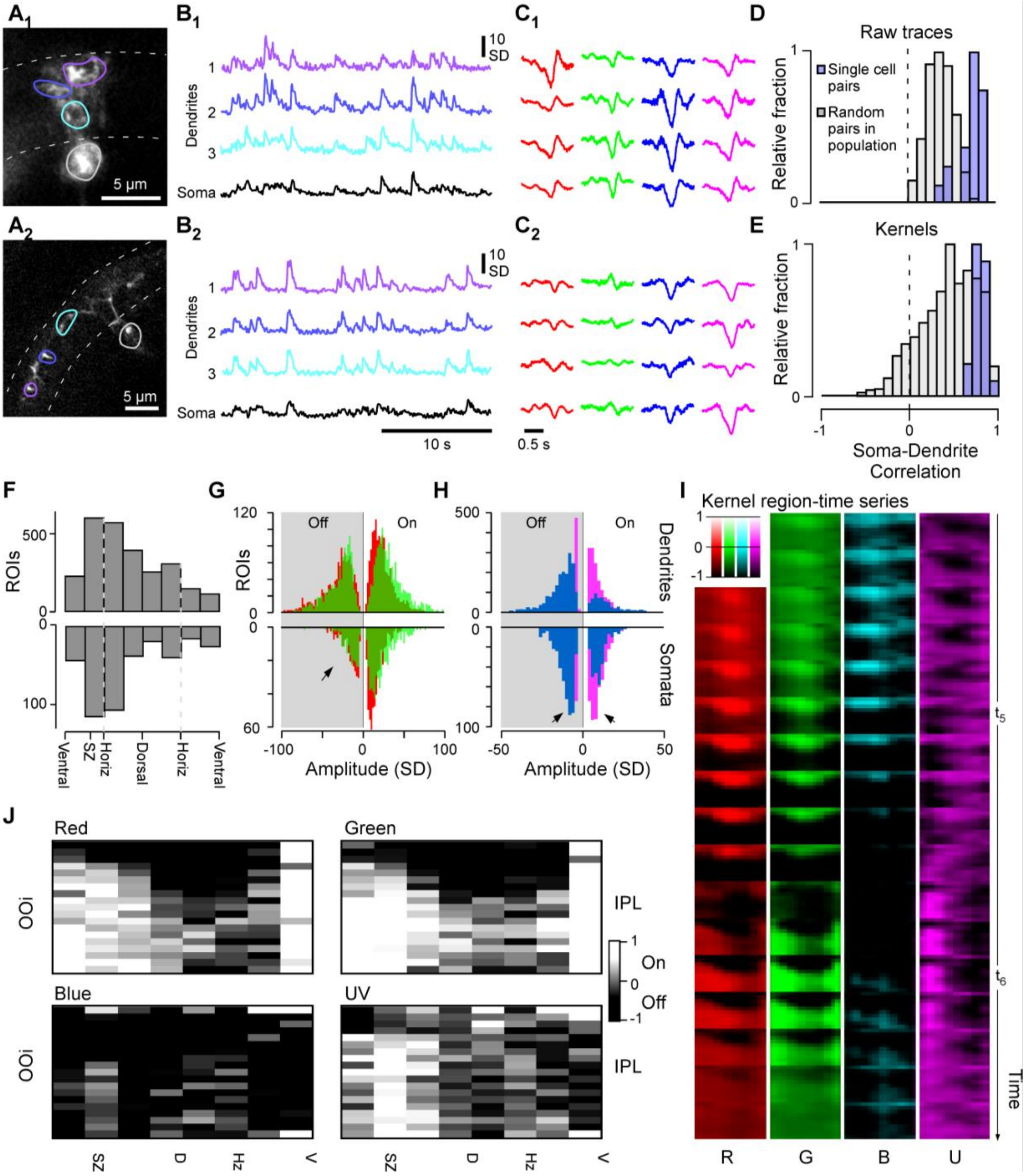

Figure S2. Linking dendritic with somatic data and eye-wide overview of RGC functions. Related to Figure 2. A-C, correlations of mGCaMP6f signals and spectral kernels from pairs of soma and dendrites belonging to the same cell, achieved by a transient expression strategy (Methods). Two RGC examples with distinct morphologies (A_1-C_1 small-field and diffuse from SZ; A_2-C_2 widefield and narrow from nasal retina) are highlighted: A, High resolution 2P scan of individual RGCs in the live eye with IPL boundaries and manually placed ROIs highlighted. B, example activity traces from each of these ROIs to naturalistic white-noise stimulation (cf. Fig S1E) and C, spectral kernels extracted from each ROI. Note that by by and large, dendritic and somatic responses (B) and kernels (C) from the same cell are very similar. **D, E**, quantification of correlations in pairs of somatic and dendritic ROIs (light purple) of raw activity traces (D) and of kernels (E). Data from n = 7 single RGCs with a total of 20 soma-dendrite pairs is shown. For comparison, the same analysis was performed for n = 400 random within-scan pairs of somatic and dendritic ROIs from n=4 nasal/dorsal population scans comprising n = 24, 16, 7, 13 somatic and n = 99, 69, 69 and 42 dendritic ROIs, respectively (light grey). In both cases, single-cell pairs were significantly more correlated than random population pairs ($p<0.001$ Wilcoxon Rank Sum test, 1 tailed), indicating that in general dendritic responses provide a useful - albeit not perfect - approximation of somatic responses. **F**, Number of dendritic (top) and

somatic (bottom, y-flipped) ROIs recorded across different positions in the eye. The relative abundance of SZ-ROIs is in line with the increased RGC numbers and thicker retinal layers [20] in this part of the eye (cf. Figure 7A). **G, H**, Distribution of dendritic (top) and somatic (bottom) On- and Off-kernels (as in Figure 2A), divided into the four wavebands, with red/green (G) and blue/UV shown together (H). Chi-Squared with Yates correction for On:Off distributions dendrites vs. somata: $p < 0.00001$ in all cases except blue, where $p = 0.0018$. **I**, Eye-IPL maps for R/G/B/U kernels (cf. Figure 2J) plotted over time (cf. Video S4) **J**, Projection of an On-Off index (OOi, Methods) in the four wavebands (as in Figure 2J) into an Eye-IPL map, with lighter and darker shades indicating an overall On- and Off- bias, respectively (see also Figure S2G,H).

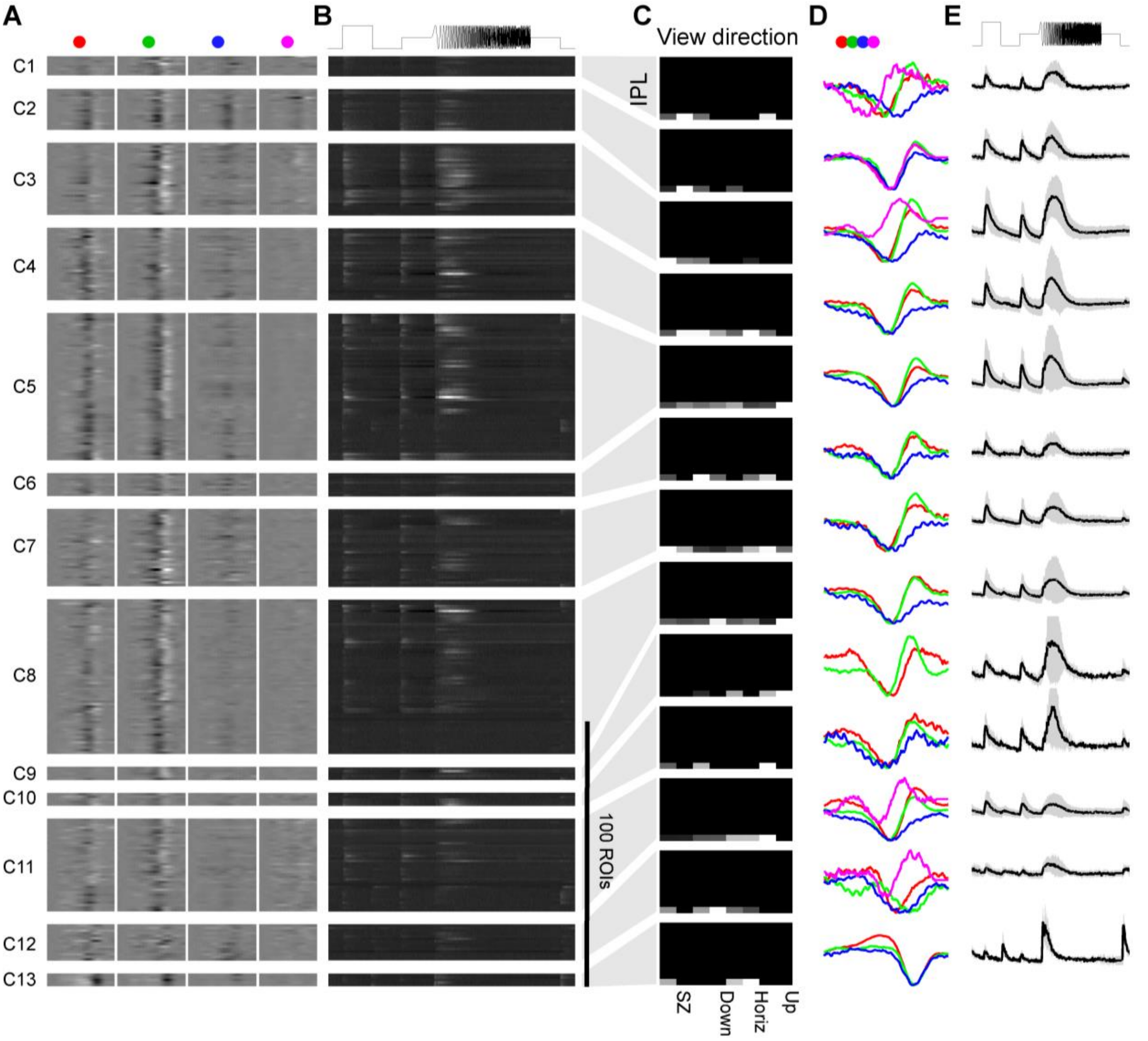

Figure S3. Functional clustering of somatic ROIs across the eye. Related to Figure 4. A-E, Somatic data from across the eye clustered based on spectral kernels, presented following the same organisation as used for dendritic data (Figure 4A-F).

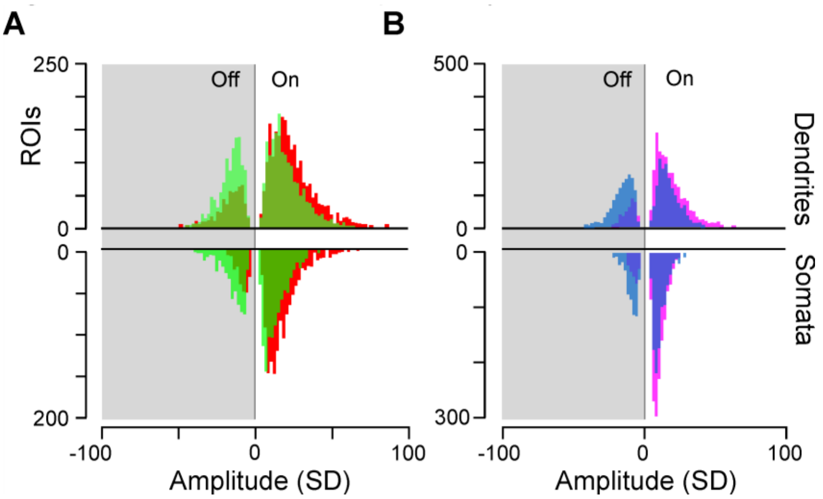

Figure S4. SZ On- and Off-responses by waveband. **Related to Figure 5. A, B**, Distribution of dendritic (top) and somatic (bottom) On- and Off-kernels (as in Figure 5B), divided into the four wavebands, with red/green (A) and blue/UV shown together (B). Dendrites n = 2,103; 1,528; 1,294; 1,864 R/G/B/U On, n = 483; 913; 984; 219 R/G/B/U Off, respectively; Somata n = 1,385; 1,034; 1,015; 1,356 R/G/B/U On, n = 236; 582; 569; 209 R/G/B/U Off, respectively. Chi-Squared with Yates correction for On:Off distributions dendrites vs. somata: p = 0.0006; 0.37; <0.00001; 0.0097 for R/G/B/U, respectively.

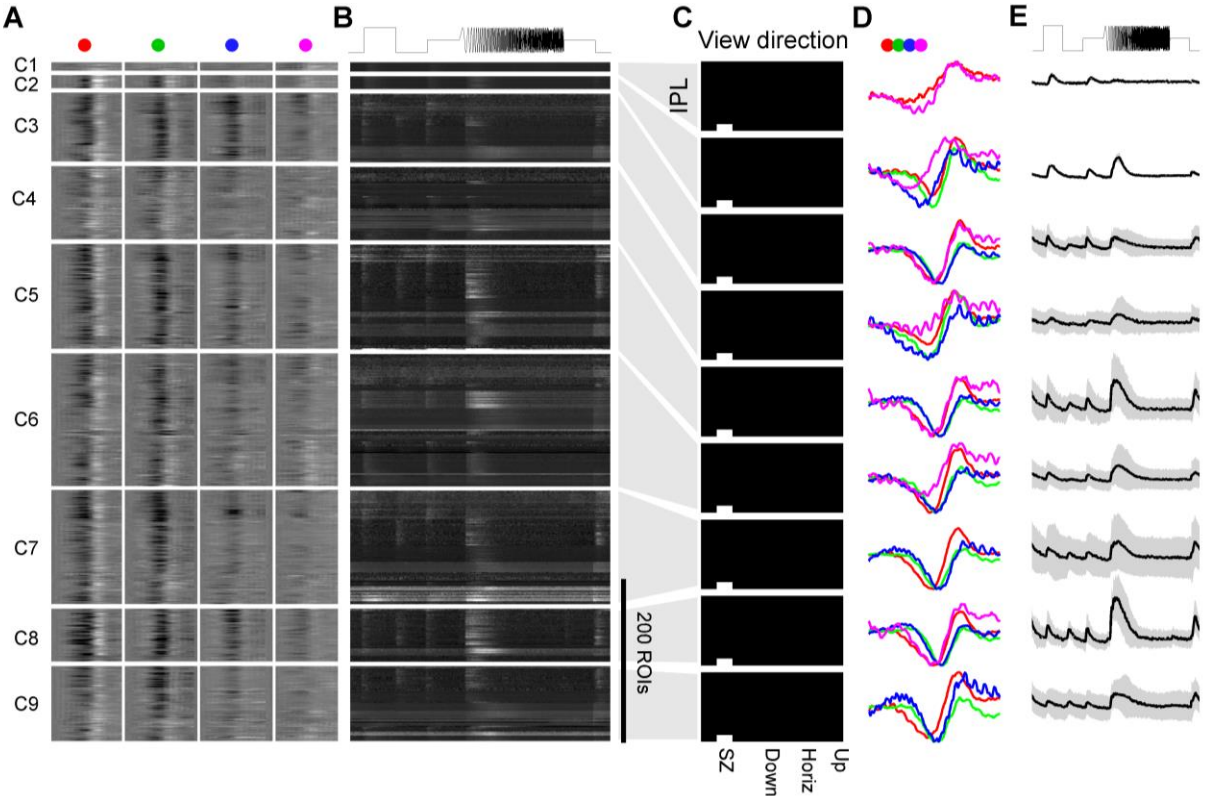

Figure S5. Functional clustering of somatic ROIs in the strike zone. **Related to Figure 6. A-E**, Somatic data from the SZ based on spectral kernels, presented following the same organisation as used for dendritic data (Figure 6A-F).

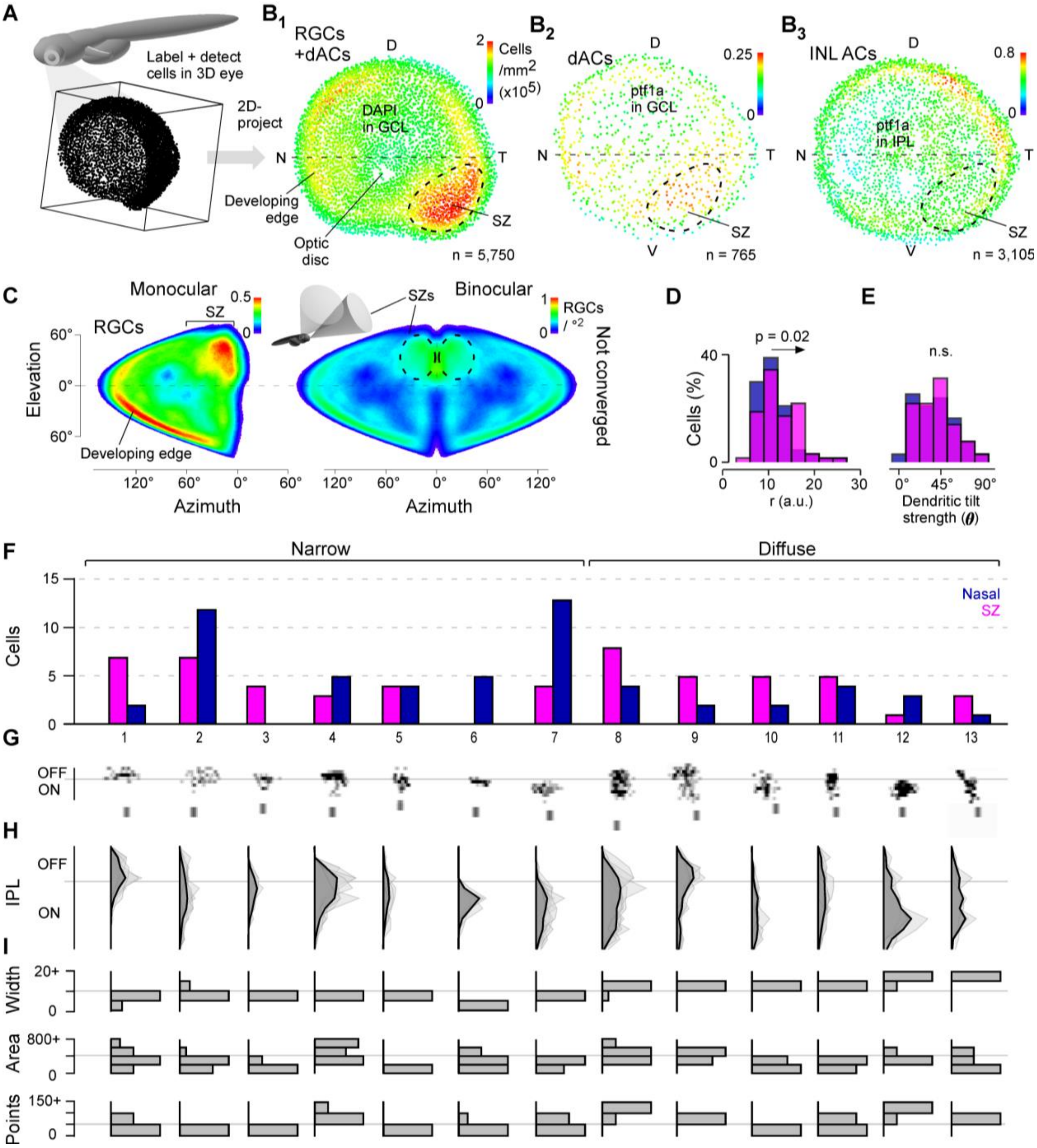

Figure S6. Further analysis on RGC anatomy. Related to Figure 7. A, Schematic of larval zebrafish and enlarged 3D representation of GCL nuclei in the eye. **B$_{1-3}$**, 2D projections of detected soma positions across the eye of all GCL cells based on a DAPI stain which includes all RGCs and all dACs (1) and selective isolation of amacrine cells in the GCL (dACs , 2) and INL (ACs, 3) based on ptf1a labelling. N = 1 eye. **C**, Projections of RGC densities into monocular (left) and binocular (right) visual space during 'rest (eyes not converged). **D,E**, Summary histograms of the distributions of r and φ (cf. Figure 7G) for quantifying dendritic tilt in photo labelled RGCs, pink: SZ, purple: Nasal. The distribution of r was weakly but significantly right shifted in SZ RGCs relative to nasal RGCs (D), while the corresponding distributions of φ were non-statistically distinct (E). Both: Two-sample Kolmogorov-Smirnov test. **F-I**, summarises an asymmetric distribution of anatomical RGC types across the eye. Photoconverted and processed RGCs from both nasal and SZ (cf. Figure 7D-M) were jointly clustered based on morphological criteria (Methods). **F**, Number of RGCs for SZ (pink/ left) and nasal retina (blue/right) allocated to each of n = 13 clusters. **G**, Individual RGC morphologies representative for each cluster. Note that each morphology's depth profile (y) is stretched five-fold relative to its lateral

spread (x) to highlight stratification differences between clusters. **H**, Mean (dark) and individual depth profiles (light) and **I**, distribution of widths, dendritic field area and number of puncta for each cluster. Clusters were divided into narrow (left) and diffusely stratified (right) based on their mean widths (I, top, cf. labels in F).
