# Supplementary material for: Zebrafish Retinal Ganglion Cells Asymmetrically Encode Spectral and Temporal Information across Visual Space
Source: Curr Biol. 2020 Aug 3;30(15):2927–2942.e7. doi: 10.1016/j.cub.2020.05.055 (PMC7416113; doi:10.1016/j.cub.2020.05.055)
Supplement: Document S2. Article plus Supplemental Information [file mmc6.pdf]

Article

Current Biology

Zebrafish Retinal Ganglion Cells Asymmetrically Encode Spectral and Temporal Information across Visual Space

Graphical Abstract

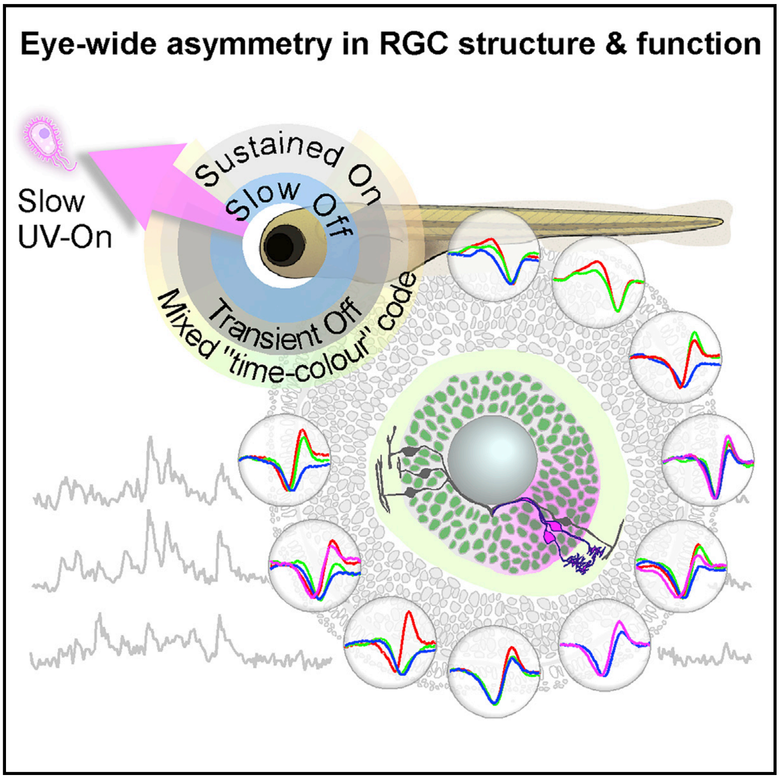

Highlights

- The structure and function of larval zebrafish RGCs are highly position dependent
- The acute zone is dominated by broadly stratifying slow UV-On cells
- Approximately half of all RGCs display diverse forms of spectral opponency
- Much spectral opponency is driven by a pervasive slow blue-Off system

Authors

Mingyi Zhou, John Bear,
Paul A. Roberts, Filip K. Janiak,
Julie Semmelhack,
Takeshi Yoshimatsu, Tom Baden

Correspondence

t.baden@sussex.ac.uk

In Brief

Zhou, Bear et al. chart the functional diversity of retinal ganglion cells in larval zebrafish by imaging responses to spectrally naturalistic full-field stimuli in the *in vivo* eye. This reveals substantial regionalization, including a UV dominance in the acute zone, and the presence of a pervasive, spectrally opponent slow blue-Off system.

Zhou et al., 2020, Current Biology *30*, 2927–2942
August 3, 2020 © 2020 The Author(s). Published by Elsevier Inc.
https://doi.org/10.1016/j.cub.2020.05.055

Current Biology

CellPress
OPEN ACCESS

Article

Zebrafish Retinal Ganglion Cells Asymmetrically Encode Spectral and Temporal Information across Visual Space

Mingyi Zhou,[1,4] John Bear,[1,2,4] Paul A. Roberts,[1] Filip K. Janiak,[1] Julie Semmelhack,[2] Takeshi Yoshimatsu,[1] and Tom Baden[1,3,5,6,*]

[1]School of Life Sciences, University of Sussex, Brighton BN19QG, UK
[2]Hong Kong University of Science and Technology, Hong Kong
[3]Institute for Ophthalmic Research, University of Tübingen, Tübingen 72076, Germany
[4]These authors contributed equally
[5]Twitter: @NeuroFishh
[6]Lead Contact
*Correspondence: t.baden@sussex.ac.uk
https://doi.org/10.1016/j.cub.2020.05.055

SUMMARY

In vertebrate vision, the tetrachromatic larval zebrafish permits non-invasive monitoring and manipulating of neural activity across the nervous system *in vivo* during ongoing behavior. However, despite a perhaps unparalleled understanding of links between zebrafish brain circuits and visual behaviors, comparatively little is known about what their eyes send to the brain via retinal ganglion cells (RGCs). Major gaps in knowledge include any information on spectral coding and information on potentially critical variations in RGC properties across the retinal surface corresponding with asymmetries in the statistics of natural visual space and behavioral demands. Here, we use *in vivo* two-photon imaging during hyperspectral visual stimulation as well as photolabeling of RGCs to provide a functional and anatomical census of RGCs in larval zebrafish. We find that RGCs' functional and structural properties differ across the eye and include a notable population of UV-responsive On-sustained RGCs that are only found in the acute zone, likely to support visual prey capture of UV-bright zooplankton. Next, approximately half of RGCs display diverse forms of color opponency, including many that are driven by a pervasive and slow blue-Off system—far in excess of what would be required to satisfy traditional models of color vision. In addition, most information on spectral contrast was intermixed with temporal information. Taken together, our results suggest that zebrafish RGCs send a diverse and highly regionalized time-color code to the brain.

INTRODUCTION

In vertebrate vision, all information sent from the eye to the brain is carried by the axons of retinal ganglion cells (RGCs) [1]. Classically, RGC types are thought to encode information about image features, such as the color, speed, or orientation of an edge. Through a mosaic arrangement of an RGC type across the retinal surface, this information can then be transmitted for all of visual space. However, what exactly all these features are [2] and to what extent their structure and function is truly homogeneous over the retinal surface to meet the demands of an animal's species-specific visual ecology [3–5] remains an area of active research [6]. Moreover, directly linking RGC types to specific visual behaviors remains a central challenge in vision science [6, 7].

Here, zebrafish offer a powerful tool for dissecting the form and function of retinal circuits [8]. Their excellent genetic access and largely transparent larval stage has made it possible to probe their visual circuits *in vivo* while animals were performing visual behaviors, such as prey capture [9–12] or predator evasion [13, 14]. In fact, prey-capture-like behaviors can be elicited by optogenetic activation of single neurons in a retinorecipient nucleus of the brain [10]. How do RGC signals from the eye supply these circuits?

Optical recordings of RGC axon terminals in the brain have shown that, like in mammals [15], larval zebrafish RGCs are tuned to object size [16] as well as orientation and motion direction [17], each organized into specific layers and regions of the brain, including the tectum, pretectum, and thalamus [17–19]. However, our understanding of RGC structure and function in zebrafish remains far from complete.

First, zebrafish have a large field of view that lets them simultaneously survey the overhead sky and the riverbed beneath them [20–22]. These parts of visual space have vastly different behavioral relevance, as well as distinct spatial, temporal, and spectral statistics [6, 20, 23, 24]. For efficient coding [25, 26], zebrafish should therefore invest in different sets of functional RGC types to support different aspects of vision across their retinal surface.

This is an open access article under the CC BY license (http://creativecommons.org/licenses/by/4.0/).

In agreement, both photoreceptor [27] and retinal bipolar cell functions [20] are asymmetrically distributed across the eye and feature pronounced reorganizations in the *area temporalis* (dubbed strike zone [SZ]) [20], which is used for visual prey capture [9, 21, 22, 27–30]. In contrast, data on functional retinal anisotropies in larval zebrafish RGCs remain outstanding (but see [18]).

Second, optically characterizing RGC functions by recording the signals of their axonal arborizations in the brain is limited by the fact they are densely packed [17] and that they are potentially subject to central presynaptic inputs [31, 32].

Third, most investigations into the function of zebrafish visual circuits have relied on long-wavelength-light stimulation to limit interference with fluorescence imaging systems [8]. However, zebrafish have tetrachromatic color vision [33] that builds on spectrally diverse retinal circuits [20, 33–35]. Wavelength is strongly associated with specific behaviors in zebrafish, including long-wavelength-dominated optomotor circuits [36] and short-wavelength-dominated prey-capture circuits [27]. However, how zebrafish vision builds on signals from spectrally selective RGC circuits is unknown.

To address these major gaps in knowledge, we imaged light-driven signals from RGCs directly in the *in vivo* eye. By "bending" the imaging scan plane to follow the natural curvature of the live eye [37] and synchronizing the stimulation light with the scanner retrace [38, 39], we chart the *in vivo* functional diversity of larval zebrafish RGCs in time and wavelength across visual space.

We find that zebrafish RGCs support a broad range of both achromatic and chromatic functions and display a notable interdependence of temporal and spectral signal processing. Moreover, the structure and function of RGCs varied strongly with position in the eye, including a regional prominence of UV-sensitive circuits in the SZ. Together, our data strongly suggest that functionally and morphologically distinct types of RGCs occupy distinct parts of the zebrafish eye to serve distinct visual functions and point to the existence of a set of specialized sustained UV-On "prey-capture RGCs" in the SZ.

RESULTS

Highly Diverse Light-Driven Responses of RGCs in the Live Eye

To record light-driven activity from RGC processes in the eye, we expressed a membrane-tagged variant of GCaMP6f (mGCaMP6f) under the RGC-associated promoter Islet2b [40]. This reliably labeled most RGCs (Figures 1A and S1A–S1C; STAR Methods). For stimulation, we presented full-field light modulated in time and wavelength based on four LEDs that were spectrally aligned with the sensitivity peaks of the zebrafish's four cone opsins (R, G, B, and UV) [20]. The power of each LED was adjusted to follow the relative power distribution across wavelength of daytime light in the zebrafish natural habitat [20, 23] to yield a "natural white": red (100%), green (50%), blue (13%), and UV (6%; Figure 1B). This adjustment ensured that RGC's spectral responses were informative about their likely performance in a natural setting. Remarkably, although high-UV power stimulation clearly affected the overall waveforms of RGC responses to noise stimulation, this resulted in no significant difference in the amplitudes and distributions of spectral receptive fields (Figures S1D–S1G).

Animals were imaged under two photon at 6–8 days post fertilization (dpf). All recordings were performed in the eye's sagittal plane (Figure 1C). In each case, after zooming in, we "bent" the scan to follow the curvature of the eye (Figure 1D, "banana scan"; STAR Methods). This allowed recording both the inner plexiform layer (IPL) and ganglion cell layer (GCL) without sampling adjacent dead space (Figure 1E) and effectively "unbent" the natural curvature of the eye, thus facilitating analysis (STAR Methods): an example 15.6-Hz recording at 64 × 32 pixel resolution comprised a "straightened" IPL in the upper part of the image and the GCL in the lower part (Figures 1E and 1F; Video S1). Together, this allowed sampling both RGC dendrites, which integrate inputs from bipolar cells (BCs) and amacrine cells (ACs) (IPL) [15], and RGC somata, whose activity is expected to largely reflect the spiking activity for transmission to the brain (GCL) [2] (STAR Methods). Throughout, we present data recorded from these distinct structures together (Figures 1G and 1H), with summary panels showing dendrites plotted on top and somata plotted on an inverted y axis below (Figures 2A and 2B). As verified using single-cell recordings (Figures S2A–S2E), and with exceptions noted below, the types and distributions of dendritic and somatic functions tended to be largely in line with each other.

For each scan, we presented two stimuli: a "natural-white" time-varying chirp stimulus [2] to assess RGCs' achromatic response properties and a 6.4-Hz natural-power-spectrum tetrachromatic binary noise stimulus to probe their spectral tuning [20]. Reverse correlation of each region of interest's (ROIs') response to this stimulus allowed computing four linear kernels, one for each stimulated waveband (STAR Methods).

In an example recording, a selection of ROIs revealed a rich diversity of response properties across both RGC dendrites and somata (Figures 1G and 1H). For example, dendritic ROI 1 was a blue-biased transient Off-process, while immediately adjacent ROI 2 was a "red versus green/blue" color opponent sustained On-process. Similarly, also different RGC somata responded in diverse manners: ROI 6 exhibited a red-dominated transient On response with a band-pass response in the frequency domain, while ROI 7 was a largely achromatic On cell. We next systematically recorded RGC responses to these stimuli across different positions in the eye.

RGCs' Polarities and Spectral Response Properties Vary across Visual Space

In total, we recorded 72 such fields of view (n = 17 fish) and automatically placed ROIs on functionally homogeneous processes based on local response correlation during the tetrachromatic noise stimulus [41] (Figures S1H–S1J; STAR Methods). Each ROI was categorized as from either dendrite or soma based on its vertical position in the scan. This yielded 2,851 dendritic and 796 somatic ROIs, of which 2,414 (84.7%) and 411 (51.6%), respectively, passed our response quality criterion (STAR Methods). ROIs from the SZ were relatively overrepresented (Figure S2F), in line with retinal thickening in this part of the eye [20, 42].

From here, low-amplitude ROIs were discarded (STAR Methods) and thereafter classed as either dominant "On" or "Off" based on the dominant sign of their largest amplitude kernel (Figure 2A; STAR Methods). Under this set of criteria,

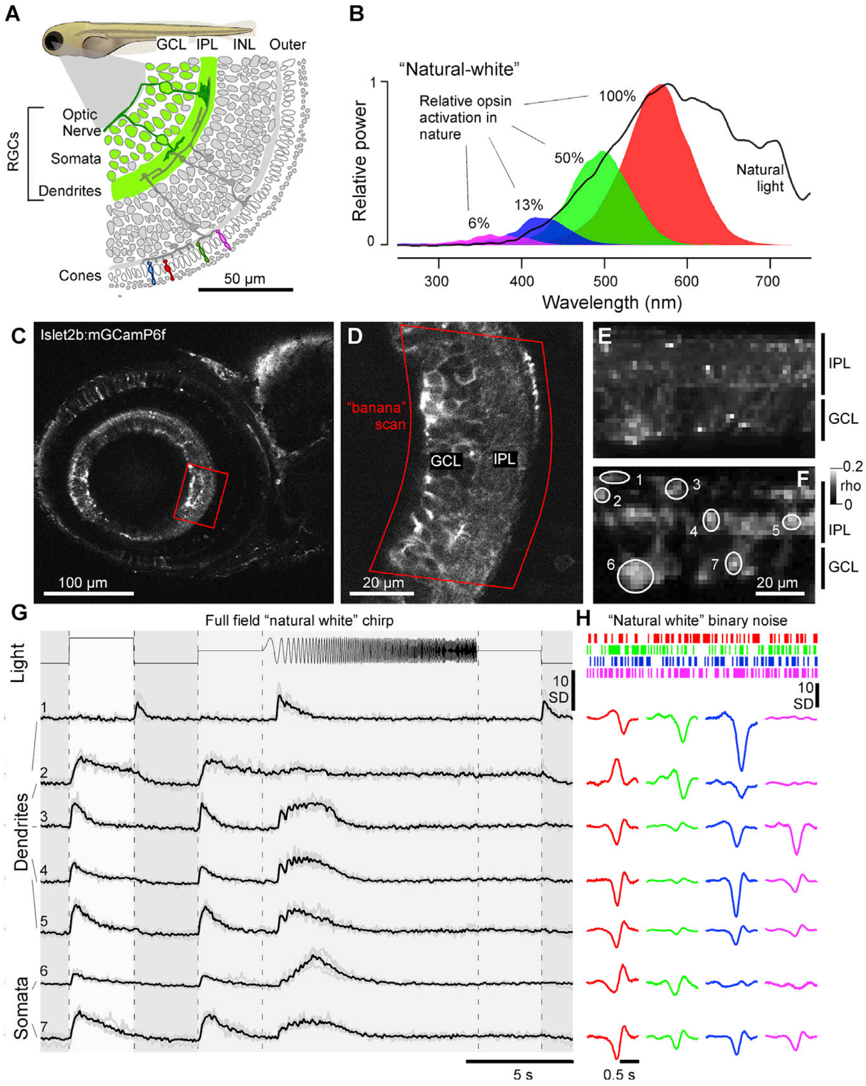

Figure 1. Recording from RGC Dendrites and Somata *In Vivo*

(A) Schematic of Islet2b:mGCaMP6f expression in RGCs (green) across a section of the larval zebrafish eye, with somata in the ganglion cell layer (GCL) and dendrites in the inner plexiform layer (IPL); see also Figures S1A–S1C. INL, inner nuclear layer.

(B) Average spectrum of natural daylight measured in the zebrafish natural habitat from the fish's point of view along the underwater horizon (solid line). Convolution of the zebrafish's four cone action spectra with this average spectrum (shadings) was used to estimate the relative power each cone surveys in nature, normalized to red cones (100%). Stimulation LED powers were relatively adjusted accordingly (“natural white”).

(C and D) GCaMP6f expression under two-photon surveyed across the entire eye's sagittal plane (C) and zoom-in to the strike zone as indicated (D). Within the zoomed field of view, a curved scan path was defined (“banana scan”) to follow the curved GCL and IPL for activity recordings (E), which effectively “straightened” the natural curvature of the eye.

(E and F) Example activity scan with RGC dendrites occupying the top part of the scan in the IPL and somata occupying the bottom part in the GCL as indicated (E) and correlation projection [41] of activity following white noise stimulation highlighting responding regions in the scan alongside example regions of interest (ROIs) (F; see also Video S1).

(G) Mean (black) and individual repeats (gray) example responses of ROIs from (E) to full-field stimulation as indicated.

(H) As (G), now showing linear kernels to red, green, blue, and UV components recovered from natural white noise stimulation (STAR Methods). Note that several ROIs display a robust UV component despite the ~20-fold attenuated stimulation power in this band relative to red (B). See also Figures S1D–S1G.

dendritic ROIs were approximately evenly (54:46 On:Off) divided into the On and Off groups (n = 1,461 On, 1,255 Off), while somata comprised relatively more On ROIs (66:34 On:Off; n = 388 On, 198 Off). Similarly, when considering only red or green kernels individually, On dominated at the level of somata (red: 65% On: n = 378 On, 208 Off; green 85% On: n = 416 On, 70 Off), but not dendrites (red: 47% On: n = 1,291 On, 1,452 Off; green: 43% On: n = 1,164 On, 1,552 Off; Figure S2G). In contrast, both at the level of somata and dendrites, blue kernels were strongly Off biased (somata: 67% Off: n = 196 On, 390 Off; dendrites: 73% Off: n = 732 On, 1,984 Off), although UV somatic, but not dendritic, kernels were On biased (somata: 64% On: n = 378 On, 211 Off; dendrites: 44% On: n = 1,192 On, 1,542 Off; Figure S2H).

Next, we computed how On- and Off-type responses in each waveband varied across the eye and thus across corresponding position in visual space (Figure 2B). This revealed that, across both dendrites and somata, On and Off processes were generally biased to the upper and lower visual fields, respectively, in line with our previous findings from bipolar cells [20]. However, blue-Off RGC processes dominated over blue-On processes throughout visual space. Finally, among dendrites, both On and Off UV processes mostly surveyed the upper visual field. However, UV-On processes were strongly biased to the frontal-upper visual field, while UV-Off processes approximately evenly surveyed upper visual space without any obvious bias for the frontal visual field. Notably, unlike other major eye-wide trends (above), the highly asymmetrical distribution of dendritic UV signals was only approximately mirrored at the level of somata. To what extent dendrite-soma differences can be explained by putative-type-specific diversity in somatic calcium channels and/or “real” differences between these distinct cellular compartments remains unclear (STAR Methods). We next asked how these spectral and regional differences are established within the layers of the IPL.

RGC Dendrites Simultaneously Encode Contrast, Time, and Color

To determine the dominant functional properties of RGC processes in different parts of the eye, we mapped each dendritic

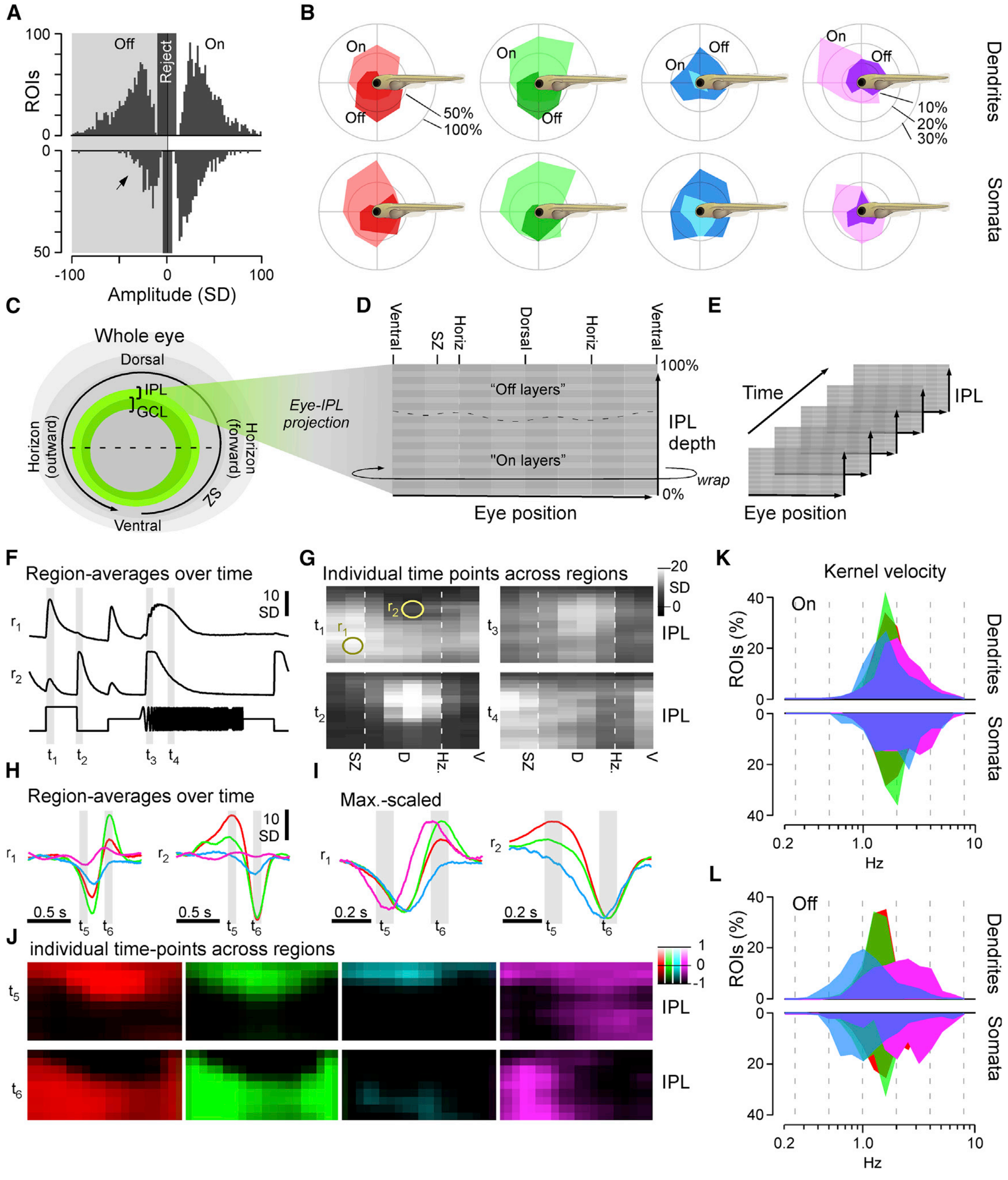

Figure 2. Major Functional Response Trends across the Eye

(A) Kernel amplitudes of all dendritic (top) and somatic (bottom; y-flipped) ROIs, shown for the maximal amplitude kernel of each ROI irrespective of color. For a breakdown by color, see Figures S2G and S2H. The arrowhead emphasizes a relative reduction in OFF responses at the level of somata. Chi-square with Yates correction for On:Off distributions dendrites versus somata: $p < 0.00001$.

(B) Prominence of different color and polarity responses among dendrites (top row) and somata (bottom row), plotted across visual space. In each case, all kernels that exceeded a minimum amplitude of 10 SDs were included. Scale bars in percent of dendritic/somatic ROIs that were recorded in a given section of the eye such that the percentages of On, Off, and non-responding (<10 SD) add to 100% are shown.

(C–E) Schematic illustrating how dendritic ROIs from different parts of the eye and IPL depth (C) were mapped into a 2D "Eye-IPL" map (D), which can then also be analyzed over time (E). Note that this involved "cutting" the circular range of eye positions such that the ventral retina is represented at either edge along the 2-projections' x axis.

(F and G) Example snapshots of mean responses to chirp stimulation (cf. Figure 1G) mapped into an eye-IPL map as schematized above (C–E). Data can be plotted as time traces for a given region of the eye and IPL (F; $r_{1,2}$ as indicated in G) or alternatively as a time-frozen snapshot of activity across the eye and IPL at different points in time (G; t_{1-4} as indicated in F). See also Videos S2 and S3.

(legend continued on next page)

ROI to a bin within an "Eye-IPL map." In this representation, the x coordinate denotes position across the eye (dorsal, nasal, etc.), while the y coordinate represents IPL depth (Figures 2C and 2D). We then computed each Eye-IPL bin's mean light response to the chirp stimulus and projected its time axis into the third dimension to yield an array linking eye position (x), IPL position (y), and time (z) (Figure 2E). In this representation, the spatially resolved mean response of all RGC dendrites could be visualized as a movie (Videos S2 and S3). Alternatively, the mean RGC response in an eye region could be displayed as a trace over time (Figure 2F) or individual time points could be displayed as images over Eye-IPL space (Figure 2G). This analysis revealed that polarity, transience, and frequency tuning of RGC dendrites all varied systematically across the eye.

For example, a region in the SZ's On layer (region 1 [r_1]) on average responded to the onset of a flash of light and exhibited broad frequency tuning during temporal flicker (Figure 2F, top). In contrast, a region within the dorsal eye's Off layer (r_2) on average exhibited an Off-dominated transient On-Off response and low-pass tuning to temporal flicker (Figure 2F, bottom). Vice versa, inspection of individual time points ($t_{1–4}$) revealed a strong asymmetry in the distribution of these response properties across both the IPL (y) and the eye (x; Figure 2G). For example, rather than forming two straight horizontal bands of On and Off responses, the position of the On-Off boundary varied strongly across the eye ($t_{1,2}$ in Figure 2G). Off responses dominated much of the IPL dorsally but were compressed to a mere ~10% of IPL width ventrally. Also, the mean temporal frequency preference varied across the eye: the dorsal-most retina exhibited the most low-pass tuning to temporal flicker, while increasingly ventral regions progressively used band-pass tuning ($t_{3,4}$ in Figure 2G; Video S3). In this achromatic regime, different parts of the eye therefore on average differentially encoded the polarity and speed of visual stimuli.

We next asked how these properties were linked to the zebrafish's four spectral input channels. For this, we mapped the spectral kernels into the same reference frame. This yielded four kernel movies, one each for red, green, blue, and UV stimulation (Video S4). We first compared the temporal profiles across the same regions r_1 and r_2 as before. In line with the achromatic chirp response (Figure 2F), r_1 was dominated by On kernels, while r_2 was dominated by Off kernels (Figures 2H and 2I). However, in each case, time courses varied greatly between spectral bands. For example, r_1 exhibited a biphasic UV-On kernel, temporally offset biphasic On kernels in red and green, and a monophasic blue Off kernel. Similarly, r_2 exhibited three distinct temporal profiles across red (biphasic), green (weakly biphasic), and blue (monophasic). Accordingly, spectral information was not only encoded through variations in gain and polarity of RGC responses but was in addition mixed with temporal information.

To more systematically explore how wavelength and time information interplay, we plotted the kernel movies as a time series (Figure S2I; cf. Video S4) and specifically highlighted the two time points that aligned with the peaks of most kernels' On and Off lobes (t_6 and t_5, respectively, in Figure 2J). In this representation, the red and green kernel maps were highly reminiscent of the achromatic On (t_1) and Off (t_2) response profiles during chirp stimulation (Figure 2J; cf. $t_{1,2}$ in Figure 2G). In contrast, blue kernels consistently lacked a dominant On lobe (Figure 2J, blue, bottom), in line with their overall Off dominance (cf. Figures 2B and S2H). Finally, UV kernels were different still: in the SZ, their IPL-depth profile approximately resembled red/green kernels (Figure 2J, magenta), although in the remainder of the eye, much of the On band seen in red/green instead transitioned into a secondary UV-Off band (Figure 2J, magenta, top). To quantify the differences in the distribution of On and Off signals, we computed an On-Off index (OOi) (STAR Methods). OOis of 1 and −1 denote regions exclusively composed of On and Off kernels, respectively, although an OOi of zero denotes an equal proportion of On and Off kernels. The resultant OOi maps confirmed the differential distributions of On and Off signals seen across in the individual kernel maps (Figure S2J).

Next, we considered the temporal domain. As across Eye-IPL space, red and green maps resembled each other (Figure 2J; cf. Figure S2I). In contrast, the blue map was consistently slowed across the entire eye, although the UV map exhibited a complex temporal behavior that in addition strongly differed between the SZ and the remainder of the eye (Figure 2J; best seen in Video S4). These broad differences were also evident from the kernels' central frequencies (spectral centroid from Fourier transform; STAR Methods), irrespective of eye position (Figures 2K and 2L). Red and green kernels exhibited a narrow range of intermediate central frequencies, although blue kernels were slowed and UV kernels were sped up. These differences were particularly pronounced for Off (Figure 2L) compared to On kernels (Figure 2K).

Together, this functional overview strongly suggests that (1) information received across the four different wavebands of light is used in distinct ways to support vision and (2) its use varies across position in the visual field [6] (Discussion). To further explore how spectral information might serve zebrafish vision at the level of the retina's output, we next assessed RGC responses for spectral opponency.

An Abundance of Temporally Complex Color Opponent RGCs

When combining the signal from multiple cone pathways for output to the brain, the number of possible wiring combinations is given by the number of possible wiring states (i.e., 3: On; Off; and no connection) raised to the power of the number of cone types (i.e., 4). Accordingly, the zebrafish's four cone types could

(H–J) As (F) and (G) but instead showing mean kernels across the four spectral wavebands, where (H) and (I) are mean and max-scaled mean kernels for Eye-IPL regions $r_{1,2}$ (as in F), respectively. (J) shows each kernel's full Eye-IPL map at two time points $t_{5,6}$ as indicated in (H) and (I) (see also Figure S2I). In the color scale bar, 0 equates to the baseline of each bin's kernel and 1/−1 to their respective maximum or minimum (cf. I). See also Video S4.

(K and L) Distribution of central frequencies (STAR Methods) of dendritic (top) and somatic (bottom; inverted y axis) kernels in the four wavebands, separated into On (K) and Off (L) kernels. Wilcoxon rank-sum test, 1 tailed with correction for multiple comparisons for all pairwise comparisons between same polarity distributions of spectral centroids, is shown. Dendrites: all $p < 0.001$ except R_{Off} versus G_{Off} ($p = 0.0011$) and G_{On} versus B_{On} ($p = 0.69$). Somata: all $p < 0.001$ except R_{On} versus U_{On} ($p = 0.00101$), R_{Off} versus G_{Off} ($p = 0.033$), G_{On} versus B_{On} ($p = 0.045$), B_{On} versus U_{On} ($p = 0.064$), R_{On} versus B_{On} ($p = 0.25$), and R_{On} versus G_{On} ($p = 0.57$).

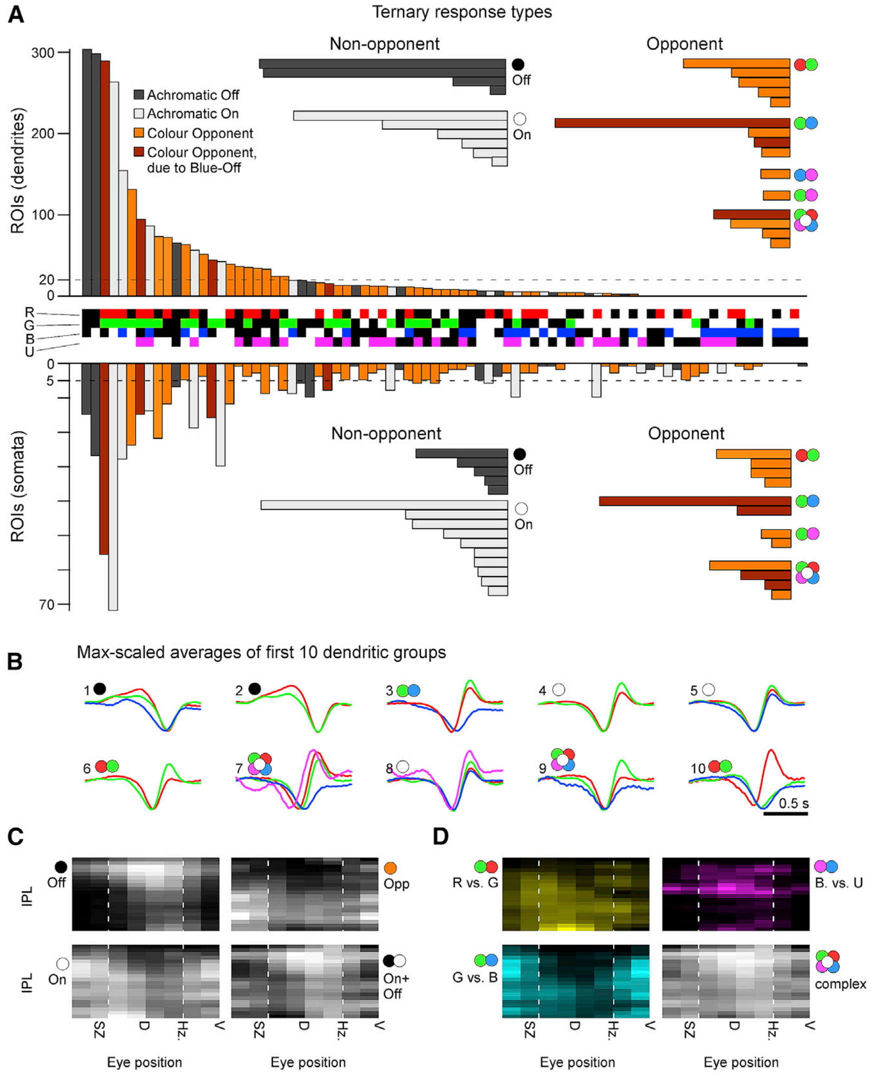

Figure 3. Diverse Color Opponencies in RGCs

(A) Each dendritic (top) and somatic (bottom; inverted y axis) ROI that passed a minimum response criterion (STAR Methods) was allocated to a single bin in a ternary classification scheme according to the relative polarities of their four spectral kernels (3 response states On, Off, and no response) raised to the power of 4 spectral channels (red, green, blue, and UV): $3^4 = 81$ possible combinations. The central row between the bar graphs indicates each bin's spectral profile: "On" (red, green, blue, and UV); "Off" (black in the respective row); and no response (white in the respective row). For example, the leftmost group, which comprised the highest number of dendritic ROIs, corresponds to ROIs displaying Off kernels in red, green, and blue, with UV showing no response. The bar graphs are color coded as follows: dark gray (non-opponent Off); light gray: (non-opponent On); and orange/brown (opponent). Brown bins indicate opponent bins that are only classified as opponent because they comprise a Blue-Off component (see main text). The horizontal insets summarize all ternary response groups that exceeded a minimum size (indicated by the dashed line) across the following categories: Off; On; and Opponents, here divided into types of spectral computations as indicated by the color circles; two-color symbols denote "simple" opponencies (single spectral zero crossing, e.g., red versus green) between the indicated wavebands (red, green, blue, and UV), although the "flower" symbol denotes complex opponencies (>1 spectral zero crossing, e.g., red and blue versus green).

(B) Maximum-amplitude scaled average kernels of the ten most abundant spectral classes among dendrites in (A).

(C and D) Dendritic groups from (A) summarized according to their position in an Eye-IPL map (cf. Figure 2). (C) summarizes major groups: Off (left, top) and On non-opponent (left, bottom); opponent (right, top); and On+Off non-opponent (right, bottom). (D) As (C), with opponent groups divided into their specific spectral computations as indicated. Note that most specific functions in (C) and (D) are restricted to specific regions of the eye and IPL. For example, green versus blue simple opponent computations occur mostly in the ON layers of the ventral retina that survey the world above the fish (D, bottom left).

be wired in a total of $3^4 = 81$ combinations. Of these, 50 are color opponent, 30 are non-opponent (15 On + 15 Off), and one represents the case where none of the four cones is functionally connected. We assessed how zebrafish RGCs span this combinatorial space and ranked the results based on the number of allocated dendritic ROIs in each wiring group (Figure 3).

Most ROIs fell into a small subset of groups with relatively simple functional wiring motifs. Among dendrites, the two most common combinations were RGB_{Off} and RG_{Off} (Figures 3A, top, dark gray, and 3B, groups 1 and 2). These non-opponent Off groups were followed by one color-opponent group (RG_{On}–B_{Off}, brown/orange; group 3) and then two non-opponent On groups (RG_{On} and RGB_{On}, light-gray; groups 4 and 5). Together, these made up 42% of all dendritic ROIs. However, subsequent groups were more diverse and largely composed of color-opponent categories to make up a total of 47% color-opponent ROIs among dendrites (e.g., Figure 3B, groups 6, 7, 9, and 10). Of these, most (75%) opponent computations had a single zero crossing in wavelength: R/G (30%), G/B (31%), B/U (8%), G/U (4%), and RU (2%), respectively (e.g., Figure 3B, groups 3, 6, and 10). The remaining 25% of opponent ROIs described diverse complex opponencies (e.g., Figure 3B, groups 7 and 9). A similar distribution of functions was found for somata (51% non-opponent and 49% opponent—of which 67% and 33% exhibited simple and complex opponencies, respectively; Figure 3A, bottom), with the notable exception of a drop in the first two Off groups (cf. Figure 2A).

As before (cf. Figure 2), the diverse functional groups of non-opponent and opponent RGC processes distributed asymmetrically across the eye and IPL depth (Figures 3C and 3D). Color-opponent RGCs existed all across the eye, but different opponencies dominated different parts of the IPL and visual field (Figure 3D). For example, B/U opponent responses were mostly restricted to the dorsal eye's Off layer, although G/B computations were mostly restricted to the ventral retina. R/G

CellPress
OPEN ACCESS

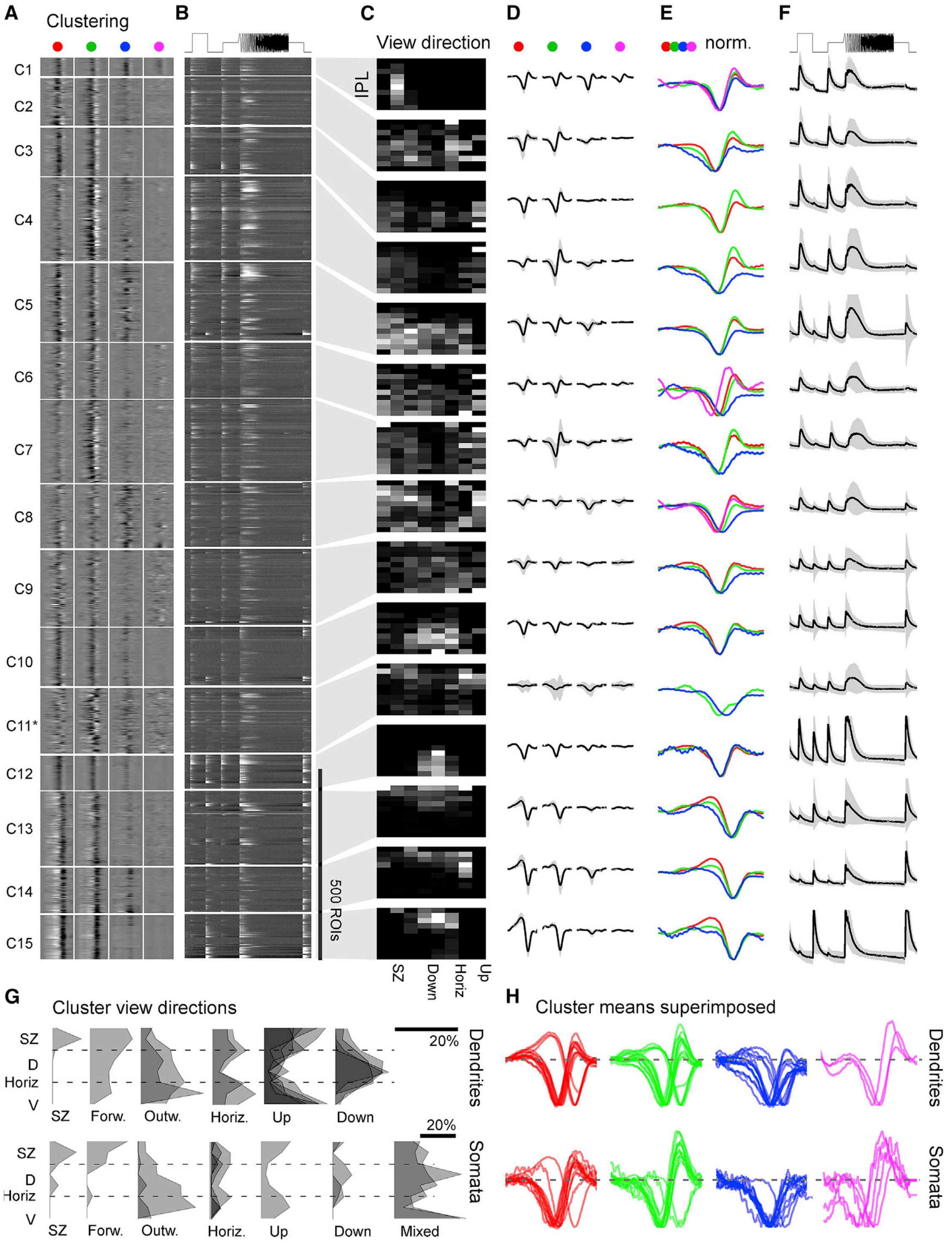

Figure 4. Functional Clustering of Dendritic ROIs

(A–F) Dendritic ROIs from across the entire eye were clustered based on their four spectral kernels (STAR Methods) to yield a total of n = 15 functional clusters that comprised a minimum of 10 ROIs. Shown are heatmaps of red, green, blue, and UV kernels (A, from left to right, respectively) and associated mean chirp response (B), with each entry showing a single ROI, followed by each cluster's Eye-IPL projection (C), each mean kernel (D), max-scaled kernels superimposed (E), and the mean chirp response (F). Error shadings in SD are shown. For clarity, low-amplitude mean kernels were omitted from column (E). Note that C_{11}* comprised a mixture of responses and may comprise a variety of low-n functional RGC types. Grayscale color maps (A–C) were linearly equalized by hand to maximize subjective discriminability of the full response range across the population of all recordings in a dataset. Lighter grays indicate higher activity/kernel amplitudes. For corresponding data on somata, see Figure S3.

(legend continued on next page)

computations were more broadly distributed but like B/U computations exhibited a preference for the dorsal retina.

Pronounced Regionalization of Functional RGC Types

Although sorting RGCs based on their relative polarities to different wavelength light is instructive to capture details in the distribution of spectral computations (Figure 3), it misses key temporal and amplitude information. As an alternative to identify the major functional RGC types of the larval zebrafish eye, we therefore turned to clustering of RGCs' full temporo-chromatic response profiles (STAR Methods). This allocated dendritic ROIs into 17 functional clusters, of which 15 ($C_{1–15}$) that contained a minimum of 10 members were kept for further analysis. Somatic ROIs instead were sorted into 20 clusters, of which 13 that contained more than 5 ROIs were kept (Figure S3). By and large, dendritic and somatic clusters exhibited similar functional properties and distributions across the eye. However, dendritic ROIs overall yielded more cleanly separated clusters, as expected based on their higher abundance and generally larger signal to noise (cf. Figure 2A). Accordingly, we here focus on the description of dendritic data, drawing on somatic clusters as a point of comparison. Importantly, whether and how our functional clusters correspond to "real" RGC types with stereotypical morphology, function, and genetics remains an open question.

Dendritic clusters included largely achromatic On ($C_{1,10,12}$) and Off ($C_{11,\ 13–15}$) clusters as well as diverse clusters that displayed a mixture of spectral and temporal response properties ($C_{2–9}$; Figure 4). However, unlike after sorting by opponency alone (Figure 3), when clustered by this wider range or response properties, opponency was a less obvious feature (though still present). Moreover, opponency was often primarily driven by the sluggish B_{Off} component opposing non-blue On kernels (Figures 4A, 4D, and 4E; see also Figure 4H). In fact, only four clusters did not exhibit an obvious sluggish B_{Off} response: C_3, which did not respond to short wavelength stimulation at all, as well as the three achromatic On clusters ($C_{1,10,12}$). Somatic clusters also showed a clear preponderance of slow B_{Off} signals ($C_{1–8,10–13}$ in Figure S3).

Most clusters of either dataset exhibited strong regional biases to only parts of visual space. For example, many dendritic clusters were biased to either the upper ($C_{4–8}$) or lower visual field ($C_{10,12–15}$; Figure 4C; summarized in Figure 4G, top). Other clusters instead showed varying degrees of bias for the horizon ($C_{2,11}$), the outward visual field ($C_{3,9}$), or the frontal visual field (C_5), including the SZ (C_1). Somatic clusters exhibited similar regional biases (Figure 4G, bottom); however, their two largest clusters ($C_{5,8}$) followed a more complex distribution, which hints at the possibility that these clusters comprised a variety of differentially distributed functional RGCs (Figure 4G, bottom, "mixed").

Among dendritic clusters, C_1 (and to a lesser extent also $C_{6,8}$) stood out in that it responded strongly to UV stimulation (Figures 4A, 4D, and 4E), despite the ~17-fold reduced signal power in our UV-stimulation light compared to red to match natural light (cf. Figure 1B). This sustained On cluster (Figure 4F) remained tightly restricted to a single regional bin, which corresponded to the SZ (Figure 4C). A functionally very similar cluster restricted to the SZ also featured among somatic ROIs (C_2). In view of the strong regionalization of behavioral responses to prey-like stimuli [22, 28], and the strong facilitatory effect of UV light in prey-capture performance [27], this suggested that dendritic C_1 and somatic C_2 comprised a subset of RGCs responsible for visual-guided prey capture in larval zebrafish [9, 10, 27]. Nevertheless, likely in part due to their extreme regional restriction, in each case, these putative prey-capture clusters only made up a tiny fraction of ROIs in this dataset (3%–5% among dendrites and somata, respectively). To therefore gain more in-depth information on the retina's output from this part of the eye, we recorded and analyzed a second functional dataset but this time restricted all recordings to the SZ (Figures 5, 6, and S4).

RGC Circuits in the Strike Zone

Following the same experimental approach as before (Figure 1), we recorded from an additional 3,542 dendritic and 1,694 somatic ROIs in the SZ (Figure 5A), of which 2,435 (68.8%) and 721 (42.6%), respectively, passed our response quality criterion (n = 87 scans, 28 fish). In line with our whole-eye data (cf. Figures 2, 3, and 4), RGCs in the SZ were strongly On biased across all wavelengths (Figure 5B), including even a slight On bias among blue responses (Figures S4A and S4B). SZ UV kernels were also generally slower compared to the remainder of the eye (Figure 5C)—in line with prolonged integration times of UV cones in this part of eye for supporting capture of UV-bright prey [27]. In agreement, SZ circuits exhibited a marked increase in the abundance of UV-On responses, which were now a dominant feature of several functional clusters (Figures 5D and 5E; dendritic $C_{1–3}$ in Figure 6; cf. Figure S5 for somatic clusters). Here, diverse RGC functions mixed UV-On components with a variety of spectral and temporal non-UV components, which in most cases resulted in a spectrally biased but broad On response. Finally, a minority (~5%) of ROIs were allocated to a single, long-wavelength biased Off cluster (C_{12}). The above features of dendritic clusters were generally mirrored among somatic clusters (Figure S5).

Not only did the SZ RGC circuits differ functionally from those observed in the remainder of the eye, they also appeared to differ in their overall anatomical distribution across the depth of the IPL: SZ-RGC clusters appearing to be more broadly stratified (Figures 6G and 6H). In fact, the SZ's only functional cluster that exhibited a narrow distribution across the IPL was the single Off cluster C_{12} (Figure 6H; cf. Figure 6C). To explore whether and

(G) Summary of cluster distributions across the eye, irrespective of IPL depths, for dendritic (top) and somatic (bottom) clusters, scaled by their relative abundance (in %; see scale bars). Eye-distribution profiles were manually allocated to one of the following groups based on which part of visual space is mainly surveyed: SZ (dendritic C_1; somatic C_2); forward (dendritic C_5; somatic C_3); outward (dendritic $C_{3,9}$; somatic $C_{9,11}$); horizon (dendritic $C_{2,11}$; somatic $C_{1,4,10}$); up (dendritic $C_{4–8}$; somatic C_7); and down (dendritic $C_{10,12–15}$; somatic $C_{12,13}$). Two large clusters (somatic $C_{5,8}$) did not obviously fit to any of these categories and were instead grouped separately as "mixed." It is possible that these clusters comprise several smaller groups of functional RGCs with distinct eye-wide distributions.

(H) As (E) for both dendritic (top) and somatic (bottom) data, but with all spectral kernels in each waveband superimposed. Note kinetic similarities across most red and green kernels and near complete absence of positive deflections in blue kernels.

CellPress
OPEN ACCESS

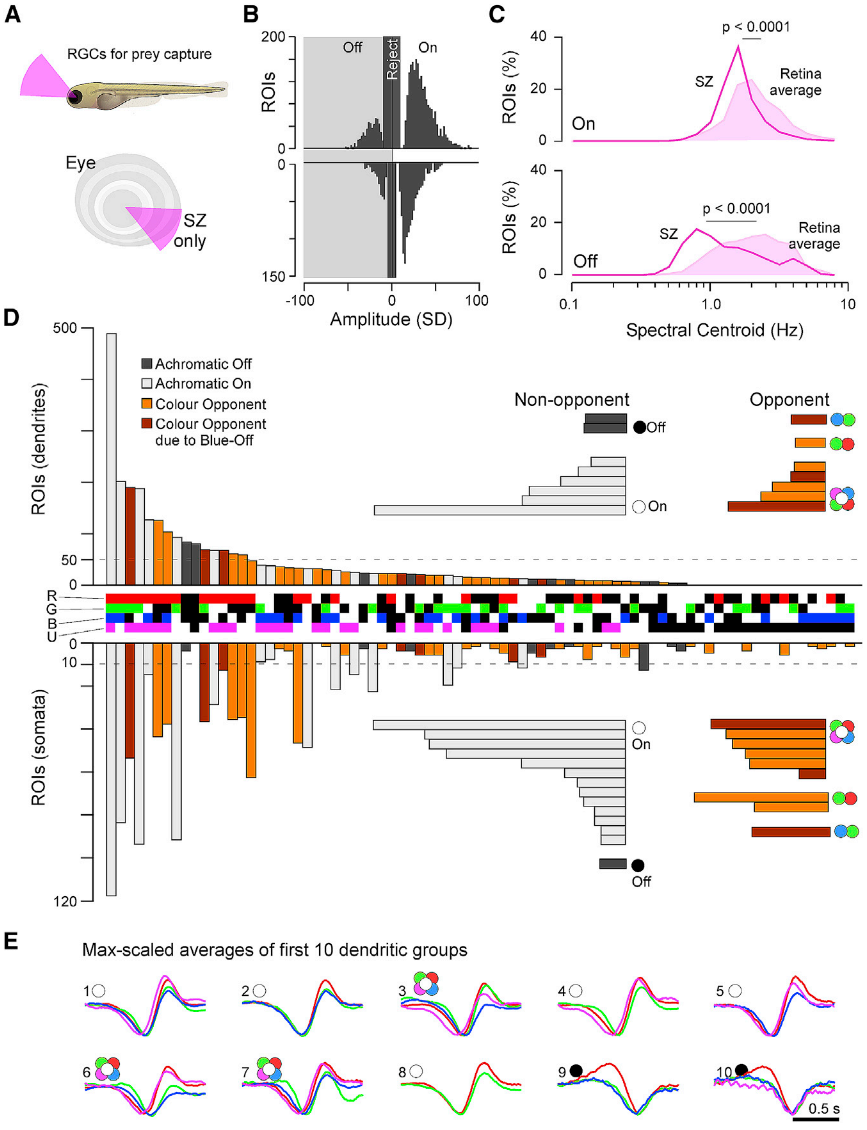

Figure 5. RGC Circuits in the Strike Zone

(A) A second series of RGC imaging experiments as shown in Figures 1, 2, 3, and 4 was performed, this time exclusively recording from the strike zone (SZ), which surveys visual space above the frontal horizon.

(B) Overview of dominant On and Off responses among dendrites (top) and somata (bottom) for the SZ. Dendrites n = 2,370 On, n = 624 Off; somata n = 1,312 On, n = 379 Off. Chi-square with Yates correction for On:Off distributions dendrites versus somata: $p < 0.22$. For details, cf. Figure 2A; for a breakdown by color, see Figures S4A and S4B.

(C) Relatively slowed central frequency tuning of SZ-UV kernels (lines) compared to the retina average of UV kernels (filled) among both On (top) and Off (bottom) kernels (cf. Figures 2K and 2L). Both $p < 0.0001$, Wilcoxon rank sum test, 1 tailed.

(D) Ternary spectral classification of SZ dataset (for details, cf. Figure 3). Overall, note the striking On dominance and increased presence of UV responses in this dataset.

(E) Maximum amplitude scaled average kernels of the ten most abundant spectral classes among dendrites in (D).

how this differential distribution of On and Off circuits is mirrored in the differential presence of distinct RGC circuits, we next assessed the anatomical distribution of morphological RGC types across the eye.

Different Morphological RGC Types Inhabit Different Parts of the Eye

In larval zebrafish, the somata of RGCs reside exclusively in the GCL, which also harbors displaced amacrine cells (dACs). To establish the number and distribution of RGCs in one intact 7-dpf eye, we labeled all somata with DAPI and identified ACs by expressing dsRed under ptfa1 [43], which is expressed in most ACs. From here, we detected all DAPI-labeled cells in the GCL (RGCs+dACs) as well as all dsRed-labeled cells in the GCL (dACs) and the inner nuclear layer (INL) (“regular” ACs) and projected each into a local distance-preserving 2D map (Figures S6A and S6B) [20]. We then subtracted dACs from GCL cells to isolate a total of n = 4,985 RGCs (Figure 7A; cf. Figures S6A–S6C; see also [18]) and summed all ptf1a-positive cells to isolate a total of n = 3,870 ACs (Figure 7B). Importantly, this approach likely overestimates the number of functional RGCs and ACs, as it includes developing cells at the retina’s edge (see also [18]). Nevertheless, as predicted from work on photoreceptors [20, 27] and in line with RGC topography in zebrafish adults [44], the density of larval RGCs was elevated in the SZ (Figure 7A; cf. projection into visual space during eye convergence: Figure 7C). In contrast, ACs were distributed approximately homogeneously (Figure 7B).

Next, we assessed the morphology of individual RGCs in different regions of the retina in an unbiased manner by expressing photoactivatable (PA)-GFP [45] in RGCs (STAR Methods). Individual GCL somata were photoconverted (Figure 7D; STAR Methods) at random in two regions of the eye: SZ and nasal retina (N). A total of n = 222 RGCs from n = 113 fish were converted and imaged. After discarding n = 3 dAC, which had no obvious axon, and another n = 88 RGCs, which were either incompletely labeled or overlapped with neighboring labeled RGCs, a final total n = 64 (SZ) and n = 67 (N) single RGCs were retained for further analysis. We then semi-automatically detected each RGC’s dendritic swellings as proxies for synaptic structures (STAR Methods) and computed their 3D location within the boundaries of the IPL, as determined after BODIPY counterstaining [18, 41]. The resultant 3D “point clouds” were used to extract morphological metrics, including the degree and direction of spatial offset between their soma and dendrites (“dendritic tilt”; Figures 7E–7H), stratification width (narrow or diffuse; Figures 7I–7K), *en face* dendritic area (Figure 7L), and

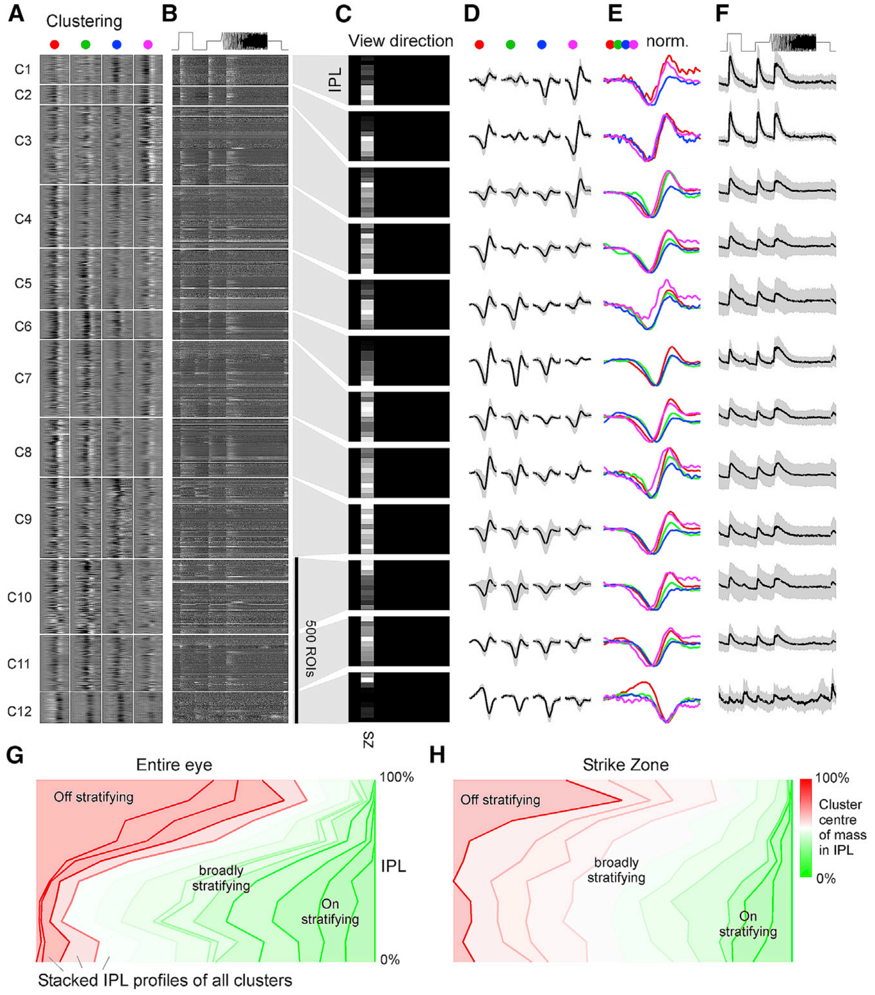

Figure 6. The SZ Is Dominated by Broadly Stratifying UV-Sensitive On Clusters

(A–F) Clustering of dendritic ROIs from SZ dataset (for details, cf. Figures 4A–4F). Note that all clusters except for C_{12} are dominated by On kernels, with $C_{1–3}$ showing pronounced UV responses despite the relatively low UV-signal power in the stimulation light (cf. Figure 1B). For corresponding clustering of SZ somata, see Figure S5.

(G and H) Side-to-side comparison of functional stratification profiles of clusters from data across the eye (G; cf. Figure 4C) and from SZ only (H; cf. C). In each case, all cluster stratification profiles of a dataset were sorted by their center of mass in the IPL (from 100%: Off to 0%: On), stacked on top of each other, and normalized to the number of ROIs per IPL depth. In addition, profiles were color coded by their center of mass in the IPL as indicated. Note that most SZ clusters (H) tended to broadly cover much of the IPL with a center of mass near the middle of the IPL (white), although eye-wide stratification profiles (G) instead showed a greater tendency to stratify in either Off (red) or On (green) layers.

number of dendritic swellings (“points”; Figure 7M). Together, this revealed systematic morphological differences between RGCs randomly sampled from the SZ and nasal retina.

First, and in contrast to the majority of known RGC types in vertebrates (e.g., [46, 47]), the dendrites of most larval zebrafish RGC were spatially offset in retinotopic space relative to the position of their soma—reminiscent of “JamB” [48] or “Mini-F-type” RGCs [49] in mice. This “dendritic tilt” consistently pointed toward the dorsal pole of the eye, resulting in retinotopically opposite tilts among nasal and SZ RGCs (Figures 7E–7H, S6D, and S6E). How this systematic asymmetry in larval zebrafish RGCs is set up developmentally—for example, by its relation to the optic fissure [50]—and whether it contributes to their function will be important to assess in the future.

Second, as predicted from our functional census (Figure 6), On-stratifying, but not Off-stratifying, SZ-RGCs tended to be more diffusely stratified across IPL depth than nasal RGCs (Figures 7I–7K), in line with the upward shift of the functional On-Off boundary and resultant “anatomical compression” of Off circuits in the SZ (cf. Figure 2 and [20]). However, in our limited sample, there was no significant difference in the distribution of RGCs’ *en face* dendritic area (Figure 7L) or numbers of dendritic swellings (Figure 7M) between the two retinal regions.

We also asked to what extent these overall stratification differences between SZ and nasal On-RGCs (Figure 7J) could be linked to the presence of distinct morphological types in different parts of the eye (Figures S6F–S6I). For this, we jointly clustered both SZ and nasal RGCs, taking into account their mean IPL depths, widths, and number of swellings (STAR Methods). This yielded 25 morphological clusters, of which 13 with a minimum of n = 4 individual members were considered for further analysis (Figure S6F). In line with a previous manually annotated census [18], RGC clusters exhibited diverse dendritic profiles, including a variety of both narrowly ($C_{1–7}$) and diffusely stratified profiles ($C_{8–13}$; Figures S6G and S6I). Here, SZ cells were approximately evenly sorted into narrow and diffuse clusters (n = 27 narrow; n = 29 diffuse); however, nasal cells were biased to narrow clusters (n = 41 narrow; n = 16 diffuse). Indeed, several individual clusters were mostly made up of RGCs coming from only one of the two retinal regions. Together, these findings tentatively suggest that distinct morphological RGC types may occupy different parts of the eye (see also [18]). In the future, it will be important to more directly assess this possibility.

DISCUSSION

We have shown that the structure, organization, and function of larval zebrafish RGC circuits depend strongly on their position in the eye—presumably to meet visuo-ecological and behavioral demands in their natural visual world [6]. The localized presence

CellPress
OPEN ACCESS

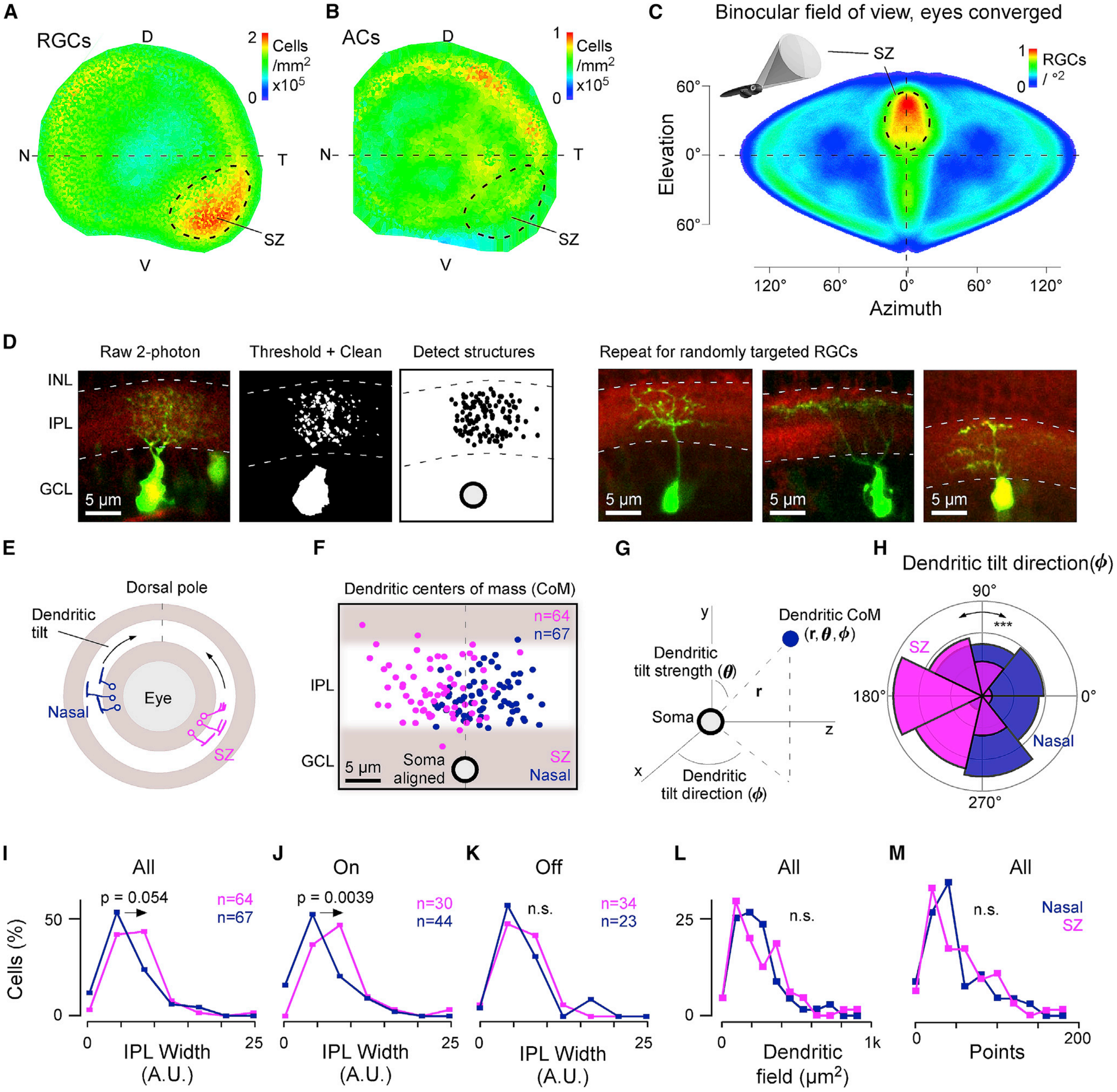

Figure 7. Elevated RGC Density and Relative Overrepresentation of Diffuse ON-RGCs in the SZ

(A and B) Density maps of all RGCs (A) and ACs (B) computed from cell counts in Figures S6A–S6C, from n = 1 retina. D, dorsal; N, nasal; SZ, strike zone; T, temporal; V, ventral.

(C) Projections of RGC densities from (A) into binocular visual space during hunting (eyes converged), as illustrated in the inset. Note that the two SZs neatly superimpose (see also [27]).

(D) Illustration of photoconversion and pre-processing pipeline for digitizing single RGC morphologies. Left: following photoconversion, cells were imaged as stacks under two-photon (green) in the background of BODIPY staining to demarcate the IPL borders (red). Cells were then thresholded and manually "cleaned" where required prior to automatic detection of image structures and alignment relative to the IPL borders. The resultant "point clouds" were used to determine summary statistics of each cell (e.g., E–M) and were also projected into density maps for visualization (Figure S6G). Right: three further examples of photo-converted RGCs are shown.

(E–M) A total of n = 64 and n = 67 randomly targeted RGCs from the SZ and nasal retina, respectively, were processed for further analysis, which included computation of their dendritic tilt (E–H), stratification widths within the IPL (I–K), *en face* dendritic field area (L), and total number of detected dendritic structures ("points"; M; STAR Methods). The dendrites of nasal (purple) and SZ (pink) RGCs both tended to tilt toward the eye's dorsal pole (E: schematic; F: soma-aligned data of all dendrites' center of mass). Dendritic tilt was quantified in soma-centered polar coordinates based on the Cartesian x,y,z coordinates that emerge from

(legend continued on next page)

of sustained UV-On RGCs in the SZ (Figures 4, 5, and 6) can be linked to their behavioral requirement to detect and localize small UV-bright prey in the upper frontal visual field [9, 22, 27, 28]. Similarly, the dominance of long- over short-wavelength responses in the lower visual field (Figure 2B) is likely related to the predominance of long-wavelength light in the lower water column [23, 51] and the zebrafish's behavioral need to monitor the ground for image shifts that drive a long-wavelength biased optomotor response [36].

In these aspects, our data from RGCs build on our previous findings on the spectral responses of presynaptic BCs [20]. However, not all functions of BCs were simply inherited by the downstream RGCs. For example, the striking dominance of slow blue-Off circuits among RGCs (Figures 2, 3, and 4) was not predicted from BCs, which instead displayed an approximately balanced mix of blue-On and Off circuits [20]. The near-complete absence of blue-On signals in zebrafish RGCs also contrasts the importance of blue-On RGC circuits in mammals [52], including in primates [53]. Next, although many of the dominant spectral opponencies observed in RGCs (Figure 3) are already present at the level of BCs [20], RGCs tended to more obviously mix time and wavelength information (Figures 2 and 4). Together, this hints at the presence of extensive further processing of temporo-spectral information beyond BCs, possibly involving ACs [54].

Surprisingly, there was no clear increase in the diversity of RGC functions (Figure 4) compared to BCs [20]—in contrast to the approximately 3-fold increase in neuron types from BCs to RGCs in mice [15]. Indeed, an anatomical census put the number of structural RGC types in larval zebrafish upward of 50 [18], far in excess of the diversity that emerged from clustering temporo-chromatic receptive fields. It is however possible, and arguably likely, that zebrafish functional RGC diversity would disproportionately increase if spatial processing were considered [55], which was not a focus of the present study. It will then also be important to address to what extent functional RGC diversity is linked to animal age.

Linking Wavelength to Visual and Behavioral Functions

In general, our data from zebrafish support the long-standing view that achromatic-image-forming vision in animals is dominated by mid- and long-wavelength channels (Figures 2G, 2J, 3A, and 3B) [56]. A close link between mid-/long-wavelength vision and achromatic vision has been discussed for diverse species of both invertebrates and vertebrates, including humans [57–59]. It allows visual systems to capitalize on the typically abundant presence of mid- and long-wavelength photons in natural light to support high spatial and temporal acuity vision carried by the majority of retinal channels [6, 59]. Spectral information can then be sent in parallel by a typically lower number of retinal output channels to "color in" the grayscale scene in central circuits [56]. Here, the finding that, in zebrafish, most opponent RGCs encode simple rather than complex opponencies is in line with previous work [20, 56, 60] and can be linked to the predominance of simple over complex spectral contrasts in natural scenes [20, 59, 61].

And yet this parsimonious textbook view remains at odds with several further observations. It does not explain (1) why nearly half of all output channels are color opponent—it should be substantially fewer [62]—(2) the striking mix of time and spectral information throughout the eye, (3) the near complete absence of blue-On circuits or the pervasive presence and general slowness of the blue-Off channel, or (4) the complex distribution of diverse UV responses throughout the eye.

Here, one explanation might relate to an implicit assumption that spectral processing and opponency should in some way link to image-forming color vision [56]. However, spectral information can be useful in additional ways. For example, opponency against blue light might also serve other non-image-forming functions, such as circadian entrainment [63], and/or serve as a depth gauge [64].

More generally, zebrafish might simply use two separate and spectrally distinct achromatic systems: one long-wavelength biased achromatic system for traditional image formatting vision and a second, short-wavelength biased achromatic system to detect image features are particularly detectable in this waveband—prey and predators. Water strongly scatters UV light, which submerges the cluttered visual background in a horizontally homogeneous UV haze. Objects in the foreground, such as nearby paramecia or predators, then stand out as UV bright or UV dark objects, respectively [27, 65]. This scatter of UV light also sets up a profound vertical brightness gradient, thus providing a reasonable explanation of why UV circuits mainly survey the upper visual field.

Such a hypothetical dual-achromatic strategy would leave the blue channel "stuck in between," encoding a mixture of red/green background and the UV foreground. As such, blue circuits could possibly provide a useful subtraction signal to better delineate achromatic red/green vision from achromatic UV vision: in the zebrafish natural habitat, daylight tends to be red/green biased but highly correlated across the full visible spectrum [23]. As a result, much of the brightness information in natural scenes will also be visible to the long-wavelength tail of the UV photopigment, thereby contaminating any UV-specific signals, which tend to be comparatively weaker [23, 27]. To a lesser extent, such spectral contamination will also occur in reverse. Here, the blue photopigment is ideally poised to help disambiguate long- from short-wavelength circuits, because it picks up the low-power tail of both signals. Accordingly, subtracting the blue component from either or both UV and red/green circuits may improve spectral delineation without strongly affecting overall signal power.

Following this line of thought, if the purpose of blue-Off circuits was not primarily to support image-forming color vision but instead to serve as a "universal background signal," we might disregard it from our account of color opponency in zebrafish RGCs (Figures 3A and 5D, highlighted in brown): in this case,

the original image stacks (G), such that *r*: distance in microns between soma and dendritic center of mass (Figure S6D), θ (0°:90°): strength of the dendritic tilt (0° and 90° denoting no tilt and maximal positive tilt, respectively; Figure S6E), and φ (0°:360°): direction of the dendritic tilt in approximately retinotopic space (approximate as the eye is curved). φ significantly differed between nasal and SZ RGCs (H). For summarizing widths, RGCs were considered as a single group (I) or split into On and Off RGCs (J and K, respectively), based on the IPL depths of their dendritic center of mass (here, the upper third of the IPL was considered "Off" and the bottom two-thirds were considered "On"). Kolmogorov-Smirnov test for circular statistics (H) and Wilcoxon rank sum test, 1 tailed (I–M), is shown.

two of the three most abundant color-opponent groups among both dendrites and somata (RG_{On}-B_{Off} and RGU_{On}-B_{Off}) would be classed as non-opponent On responses (Figure 3A). Remaining color-opponent RGCs would then drop to 28% and 32% among dendrites and somata, respectively [56].

The link between spectral and temporal processing might also be reasonably explained by a dual-achromatic strategy segregated by a blue channel: a blue-Off background subtraction system might benefit from a long integration time to be relatively less perturbed by rapid changes in the visual scene.

Notwithstanding, these ideas remain largely speculative. In the future, it will be important to specifically explore testable predictions that emerge.

The Zebrafish Area Temporalis as an Accessible Model for the Primate Fovea?

Most studies on foveal function and dysfunction have remained restricted to primates, because many accessible model systems in vertebrate vision research, notably including mice, do not feature a similar specialization [6]. However, the larval zebrafish's *area temporalis* (SZ) mimics several properties of the primate fovea and may thus serve as a potentially useful and experimentally accessible alternative. Behaviorally, larval zebrafish guide their SZ onto prey targets during fixational eye movements for high-acuity binocular vision and distance estimation [22, 27, 28], in many ways similar to fixational eye movements in primates. Functionally, zebrafish SZ UV cones boost signal to noise by using enlarged outer segments and slowed kinetics based on molecular tuning of their phototransduction cascade [27]—all specializations that also occur in primate foveal cones [4, 66]. Here, our data on RGC distributions and functions in larval zebrafish lend further credence to this notion. First, zebrafish have a fovea-like reduced ratio of ACs compared to RGCs in their SZ (Figures 7A and 7B). Second, like in the primate fovea [4, 67], SZ RGC circuits are spectrally distinct to those of the peripheral retina (Figures 2, 3, and 4), and they are also slower (Figure 5C). Third, retinal ganglion cells in the SZ are structurally distinct from those located in rest of the eye (Figures 7E–7K and S6F–S6I) and include anatomical types that have a tiny dendritic field area that barely exceeds the width of their soma (Figures S6F and S6G; see also [18]). A small dendritic field is generally associated with a correspondingly small spatial receptive field [68], which would be critical to detect small prey-like visual targets [9, 22, 27]. In the future, it will be interesting to explore what further aspects of the zebrafish SZ—if any—can be paralleled to foveal vision in primates. Moreover, it will be critical to evaluate to what extent this growing series of functional, structural, and molecular links between the two retinal systems may generalize across acute zones of other vertebrates [6].

RGCs for Prey Capture

Bringing together behavioral [9, 21, 22, 28–30], physiological [9, 10, 20, 27], and anatomical [18, 42] evidence, it seems clear that RGCs specifically in the SZ are key to several aspects of visual prey capture. Here, our RGC data show that this part of the eye is dominated by a diversity of On circuits that are biased to either short- or long-wavelength light in addition to a handful of more broadly tuned circuits. Conceptually, any or all of these might support the detection of brighter-than-background prey objects in a variety of spectral lighting conditions and might go partway to explaining why prey capture behavior and associated brain activity can occur even in the absence of UV illumination [9, 10, 22, 28] or indeed the absence of UV cones [27]. Nevertheless, in view of (1) the natural appearance of zebrafish prey items when illuminated by the sun [27]; (2) the dominance of UV signaling in the SZ, from photoreceptors [27] via bipolar cells [20] to RGCs (this study); and (3) the fact that UV-cone ablation dramatically reduces prey capture performance in both larvae [27] and adults [69], it seems likely that specifically UV-cone-driven RGC circuits are key to this behavior. In contrast, the comparatively small number of more broadly tuned Off-RGC circuits in the SZ might underlie the detection of darker-than-background objects [22], which leads to the testable prediction that, in this case, UV cones should only play a minor role in behavioral performance.

Next, prey-capture RGCs are expected to send axon collaterals to axonal arborization field 7 (AF7) [9, 10]. Here, several of our "diffuse" morphological SZ clusters (Figure S6G) were reminiscent of candidate prey-capture-RGC morphologies previously identified based on their central projections [9]. A broad stratification strategy among SZ ON circuits might be useful to integrate retinal signals across a broad range of presynaptic circuits that encode a common position in visual space. Such an arrangement might be a key requisite to build high signal-to-noise RGC circuits with small receptive fields for reliable detection of small targets during prey capture.

Taken together, it appears that we ought to be searching for potentially small-field but diffusely stratifying RGCs in the SZ that show a robust sustained On response to UV light, as well as possibly an additional On response to longer wavelength light. Serendipitously, as part of our single-cell imaging experiments, we did come across one RGC that appeared to approximate these search terms (Figures S2A–S2D). Understanding whether and how RGCs such as these contribute to visual-prey capture behavior will be an important goal in the future. In this case, it will also be critical to specifically probe responses of SZ-RGCs to spectrally naturalistic spatial stimuli [55].

STAR★METHODS

Detailed methods are provided in the online version of this paper and include the following:

- KEY RESOURCES TABLE
- RESOURCE AVAILABILITY
  - Lead Contact
  - Materials Availability
  - Data and Code Availability
- EXPERIMENTAL MODEL AND SUBJECT DETAILS
  - Animals
- METHOD DETAILS
  - Tissue preparation, immunolabeling, and imaging
  - Cell density mapping
  - Axonal tracing
  - Photoactivation
  - Two-photon functional imaging and stimulation parameters
- QUANTIFICATION AND STATISTICAL ANALYSIS
  - Data analysis

- ○ ROI placements and quality criterion
- ○ A note on ROI segmentation and identity
- ○ Kernel polarity
- ○ Digitizing photoactivated cells
- ○ Quantifying dendritic tilt
- ○ Morphology Clustering
- ○ Functional data pre-processing and receptive field mapping
- ○ Eye-IPL maps
- ○ On-Off index (OOi)
- ○ Ternary response classification
- ○ Feature extraction and Clustering

SUPPLEMENTAL INFORMATION

Supplemental Information can be found online at https://doi.org/10.1016/j.cub.2020.05.055.

ACKNOWLEDGMENTS

We thank Daniel Osorio and Katrin Franke for critical feedback, Philipp Berens for advising on statistical analyses, and Maxime Zimmermann for providing the 3D zebrafish model. The authors would also like to acknowledge support from the FENS-Kavli Network of Excellence and the EMBO YIP. Funding was provided from the European Research Council (ERC-StG "NeuroVisEco" 677687 to T.B.), the UKRI (BBSRC, BB/R014817/1 and MRC, MC_PC_15071 to T.B.), the Leverhulme Trust (PLP-2017-005 to T.B.), the Lister Institute of Preventive Medicine (to T.B.), the Marie Curie Sklodowska Actions individual fellowship ("ColourFish" 748716 to T.Y.) from the European Union's Horizon 2020 research and innovation programme, and the Sussex-HKUST joint PhD programme (to T.B. and J.S.).

AUTHOR CONTRIBUTIONS

M.Z., J.B., T.Y., and T.B. designed the study, with input from all authors; M.Z., J.B., and T.Y. generated novel transgenic lines; F.K.J. developed custom two-photon scan options; M.Z. and J.B. performed functional recordings and data pre-processing: population recordings around the eye, single cell recordings, comparison of flat versus natural stats noise stimulation (M.Z.), and population recordings in the SZ (J.B.); J.B. performed paGFP experiments and retinal cell counts and data pre-processing; T.Y. computed cell-density projections into visual space; P.A.R. performed clustering analyses and dendritic tilt quantification; and T.B. performed additional data analysis and wrote the manuscript, with input from all authors.

DECLARATION OF INTERESTS

The authors declare no competing interests.

Received: February 7, 2020
Revised: March 31, 2020
Accepted: May 18, 2020
Published: June 11, 2020

REFERENCES

1. Wässle, H. (2004). Parallel processing in the mammalian retina. Nat. Rev. Neurosci. *5*, 747–757.
2. Baden, T., Berens, P., Franke, K., Román Rosón, M., Bethge, M., and Euler, T. (2016). The functional diversity of retinal ganglion cells in the mouse. Nature *529*, 345–350.
3. Bleckert, A., Schwartz, G.W., Turner, M.H., Rieke, F., and Wong, R.O.L. (2014). Visual space is represented by nonmatching topographies of distinct mouse retinal ganglion cell types. Curr. Biol. *24*, 310–315.
4. Sinha, R., Hoon, M., Baudin, J., Okawa, H., Wong, R.O.L., and Rieke, F. (2017). Cellular and circuit mechanisms shaping the perceptual properties of the primate fovea. Cell *168*, 413–426.e12.
5. Sabbah, S., Gemmer, J.A., Bhatia-Lin, A., Manoff, G., Castro, G., Siegel, J.K., Jeffery, N., and Berson, D.M. (2017). A retinal code for motion along the gravitational and body axes. Nature *546*, 492–497.
6. Baden, T., Euler, T., and Berens, P. (2020). Understanding the retinal basis of vision across species. Nat. Rev. Neurosci. *21*, 5–20.
7. Lettvin, J.Y., Maturana, H.R., McCulloch, W.S., and Pitts, W.H. (1959). What the frog's eye tells the frog's brain. Proc. IRE *47*, 1940–1951.
8. Bollmann, J.H. (2019). The zebrafish visual system: from circuits to behavior. Annu. Rev. Vis. Sci. *5*, 269–293.
9. Semmelhack, J.L., Donovan, J.C., Thiele, T.R., Kuehn, E., Laurell, E., and Baier, H. (2014). A dedicated visual pathway for prey detection in larval zebrafish. eLife *3*, e04878.
10. Antinucci, P., Folgueira, M., and Bianco, I.H. (2019). Pretectal neurons control hunting behaviour. eLife *8*, e48114.
11. Muto, A., Lal, P., Ailani, D., Abe, G., Itoh, M., and Kawakami, K. (2017). Activation of the hypothalamic feeding centre upon visual prey detection. Nat. Commun. *8*, 15029.
12. Muto, A., Ohkura, M., Abe, G., Nakai, J., and Kawakami, K. (2013). Real-time visualization of neuronal activity during perception. Curr. Biol. *23*, 307–311.
13. Temizer, I., Donovan, J.C., Baier, H., and Semmelhack, J.L. (2015). A visual pathway for looming-evoked escape in larval zebrafish. Curr. Biol. *25*, 1823–1834.
14. Dunn, T.W., Gebhardt, C., Naumann, E.A., Riegler, C., Ahrens, M.B., Engert, F., and Del Bene, F. (2016). Neural circuits underlying visually evoked escapes in larval zebrafish. Neuron *89*, 613–628.
15. Baden, T., Schubert, T., Berens, P., and Euler, T. (2018). The functional organization of vertebrate retinal circuits for vision (Oxford Research Encyclopedia of Neuroscience). https://oxfordre.com/neuroscience/view/10.1093/acrefore/9780190264086.001.0001/acrefore-9780190264086-e-68.
16. Preuss, S.J., Trivedi, C.A., vom Berg-Maurer, C.M., Ryu, S., and Bollmann, J.H. (2014). Classification of object size in retinotectal microcircuits. Curr. Biol. *24*, 2376–2385.
17. Nikolaou, N., Lowe, A.S., Walker, A.S., Abbas, F., Hunter, P.R., Thompson, I.D., and Meyer, M.P. (2012). Parametric functional maps of visual inputs to the tectum. Neuron *76*, 317–324.
18. Robles, E., Laurell, E., and Baier, H. (2014). The retinal projectome reveals brain-area-specific visual representations generated by ganglion cell diversity. Curr. Biol. *24*, 2085–2096.
19. Gabriel, J.P., Trivedi, C.A., Maurer, C.M., Ryu, S., and Bollmann, J.H. (2012). Layer-specific targeting of direction-selective neurons in the zebrafish optic tectum. Neuron *76*, 1147–1160.
20. Zimmermann, M.J.Y., Nevala, N.E., Yoshimatsu, T., Osorio, D., Nilsson, D.-E., Berens, P., and Baden, T. (2018). Zebrafish differentially process color across visual space to match natural scenes. Curr. Biol. *28*, 2018–2032.e5.
21. Patterson, B.W., Abraham, A.O., MacIver, M.A., and McLean, D.L. (2013). Visually guided gradation of prey capture movements in larval zebrafish. J. Exp. Biol. *216*, 3071–3083.
22. Bianco, I.H., Kampff, A.R., and Engert, F. (2011). Prey capture behavior evoked by simple visual stimuli in larval zebrafish. Front. Syst. Neurosci. *5*, 101.
23. Nevala, N.E., and Baden, T. (2019). A low-cost hyperspectral scanner for natural imaging and the study of animal colour vision above and under water. Sci. Rep. *9*, 10799.
24. Parichy, D.M. (2015). Advancing biology through a deeper understanding of zebrafish ecology and evolution. eLife *4*, e05635.
25. Attneave, F. (1954). Some informational aspects of visual perception. Psychol. Rev. *61*, 183–193.

CellPress
OPEN ACCESS

26. Barlow, H.B. (1961). Possible principles underlying the transformation of sensory messages. In Sensory Communication, W. Rosenblith, ed. (MIT), pp. 217–234.
27. Yoshimatsu, T., Schröder, C., Berens, P., and Baden, T. (2019). Cellular and molecular mechanisms of photoreceptor tuning for prey capture in larval zebrafish. bioRxiv. https://doi.org/10.1101/744615.
28. Mearns, D.S., Donovan, J.C., Fernandes, A.M., Semmelhack, J.L., and Baier, H. (2020). Deconstructing hunting behavior reveals a tightly coupled stimulus-response loop. Curr. Biol. *30*, 54–69.e9.
29. Trivedi, C.A., and Bollmann, J.H. (2013). Visually driven chaining of elementary swim patterns into a goal-directed motor sequence: a virtual reality study of zebrafish prey capture. Front. Neural Circuits *7*, 86.
30. McElligott, M.B., and O'malley, D.M. (2005). Prey tracking by larval zebrafish: axial kinematics and visual control. Brain Behav. Evol. *66*, 177–196.
31. Sajovic, P., and Levinthal, C. (1983). Inhibitory mechanism in zebrafish optic tectum: visual response properties of tectal cells altered by picrotoxin and bicuculline. Brain Res. *271*, 227–240.
32. Liang, L., Fratzl, A., El Mansour, O., Reggiani, J.D.S., Chen, C., and Andermann, M.L. (2020). Selective gating of retinal information by arousal. bioRxiv. https://doi.org/10.1101/2020.03.12.989913.
33. Meier, A., Nelson, R., and Connaughton, V.P. (2018). Color processing in zebrafish retina. Front. Cell. Neurosci. *12*, 327.
34. Connaughton, V.P., and Nelson, R. (2010). Spectral responses in zebrafish horizontal cells include a tetraphasic response and a novel UV-dominated triphasic response. J. Neurophysiol. *104*, 2407–2422.
35. Klaassen, L.J., de Graaff, W., van Asselt, J.B., Klooster, J., and Kamermans, M. (2016). Specific connectivity between photoreceptors and horizontal cells in the zebrafish retina. J. Neurophysiol. *116*, 2799–2814.
36. Orger, M.B., and Baier, H. (2005). Channeling of red and green cone inputs to the zebrafish optomotor response. Vis. Neurosci. *22*, 275–281.
37. Janiak, F.K., Bartel, P., Bale, M.R., Yoshimatsu, T., Komulainen, E., Zhou, M., Staras, K., Prieto-Godino, L.L., Euler, T., Maravall, M., et al. (2019). Divergent excitation two photon microscopy for 3D random access mesoscale imaging at single cell resolution. bioRxiv. https://doi.org/10.1101/821405.
38. Euler, T., Hausselt, S.E., Margolis, D.J., Breuninger, T., Castell, X., Detwiler, P.B., and Denk, W. (2009). Eyecup scope–optical recordings of light stimulus-evoked fluorescence signals in the retina. Pflugers Arch. *457*, 1393–1414.
39. Euler, T., Franke, K., and Baden, T. (2019). Studying a light sensor with light: multiphoton imaging in the retina. In Multiphoton Microscopy, E. Hartveit, ed. (Springer), pp. 225–250.
40. Pittman, A.J., Law, M.-Y., and Chien, C.-B. (2008). Pathfinding in a large vertebrate axon tract: isotypic interactions guide retinotectal axons at multiple choice points. Development *135*, 2865–2871.
41. Franke, K., Berens, P., Schubert, T., Bethge, M., Euler, T., and Baden, T. (2017). Inhibition decorrelates visual feature representations in the inner retina. Nature *542*, 439–444.
42. Schmitt, E.A., and Dowling, J.E. (1999). Early retinal development in the zebrafish, Danio rerio: light and electron microscopic analyses. J. Comp. Neurol. *404*, 515–536.
43. Jusuf, P.R., and Harris, W.A. (2009). Ptf1a is expressed transiently in all types of amacrine cells in the embryonic zebrafish retina. Neural Dev. *4*, 34.
44. Pita, D., Moore, B.A., Tyrrell, L.P., and Fernández-Juricic, E. (2015). Vision in two cyprinid fish: implications for collective behavior. PeerJ *3*, e1113.
45. Patterson, G.H., and Lippincott-Schwartz, J. (2002). A photoactivatable GFP for selective photolabeling of proteins and cells. Science *297*, 1873–1877.
46. Bae, J.A., Mu, S., Kim, J.S., Turner, N.L., Tartavull, I., Kemnitz, N., Jordan, C.S., Norton, A.D., Silversmith, W.M., Prentki, R., et al.; Eyewirers (2018). Digital museum of retinal ganglion cells with dense anatomy and physiology. Cell *173*, 1293–1306.e19.
47. Dacey, D.M. (1999). Primate retina: cell types, circuits and color opponency. Prog. Retin. Eye Res. *18*, 737–763.
48. Kim, I.-J., Zhang, Y., Yamagata, M., Meister, M., and Sanes, J.R. (2008). Molecular identification of a retinal cell type that responds to upward motion. Nature *452*, 478–482.
49. Rousso, D.L., Qiao, M., Kagan, R.D., Yamagata, M., Palmiter, R.D., and Sanes, J.R. (2016). Two pairs of ON and OFF retinal ganglion cells are defined by intersectional patterns of transcription factor expression. Cell Rep. *15*, 1930–1944.
50. Martinez-Morales, J.R., and Wittbrodt, J. (2009). Shaping the vertebrate eye. Curr. Opin. Genet. Dev. *19*, 511–517.
51. Muaddi, J.A., and Jamal, M.A. (1991). Solar spectrum at depth in water. Renew. Energy *1*, 31–35.
52. Marshak, D.W., and Mills, S.L. (2014). Short-wavelength cone-opponent retinal ganglion cells in mammals. Vis. Neurosci. *31*, 165–175.
53. Calkins, D.J., Tsukamoto, Y., and Sterling, P. (1998). Microcircuitry and mosaic of a blue-yellow ganglion cell in the primate retina. J. Neurosci. *18*, 3373–3385.
54. Torvund, M.M., Ma, T.S., Connaughton, V.P., Ono, F., and Nelson, R.F. (2017). Cone signals in monostratified and bistratified amacrine cells of adult zebrafish retina. J. Comp. Neurol. *525*, 1532–1557.
55. Franke, K., Maia Chagas, A., Zhao, Z., Zimmermann, M.J.Y., Bartel, P., Qiu, Y., Szatko, K.P., Baden, T., and Euler, T. (2019). An arbitrary-spectrum spatial visual stimulator for vision research. eLife *8*, e48779.
56. Baden, T., and Osorio, D. (2019). The retinal basis of vertebrate color vision. Annu. Rev. Vis. Sci. *5*, 177–200.
57. Osorio, D., and Vorobyev, M. (2008). A review of the evolution of animal colour vision and visual communication signals. Vision Res. *48*, 2042–2051.
58. Solomon, S.G., and Lennie, P. (2007). The machinery of colour vision. Nat. Rev. Neurosci. *8*, 276–286.
59. Buchsbaum, G., and Gottschalk, A. (1983). Trichromacy, opponent colours coding and optimum colour information transmission in the retina. Proc. R. Soc. Lond. B Biol. Sci. *220*, 89–113.
60. Kamermans, M., Kraaij, D.A., and Spekreijse, H. (1998). The cone/horizontal cell network: a possible site for color constancy. Vis. Neurosci. *15*, 787–797.
61. Maloney, L.T. (1986). Evaluation of linear models of surface spectral reflectance with small numbers of parameters. J. Opt. Soc. Am. A *3*, 1673–1683.
62. Lewis, A., and Zhaoping, L. (2006). Are cone sensitivities determined by natural color statistics? J. Vis. *6*, 285–302.
63. Mouland, J.W., Martial, F., Watson, A., Lucas, R.J., and Brown, T.M. (2019). Cones support alignment to an inconsistent world by suppressing mouse circadian responses to the blue colors associated with twilight. Curr. Biol. *29*, 4260–4267.e4.
64. Veraszto, C., Gühmann, M., Jia, H., Rajan, V.B.V., Bezares-Calderón, L.A., Piñeiro-Lopez, C., Randel, N., Shahidi, R., Michiels, N.K., Yokoyama, S., et al. (2018). Ciliary and rhabdomeric photoreceptor-cell circuits form a spectral depth gauge in marine zooplankton. eLife *7*, e36440.
65. Cronin, T.W., and Bok, M.J. (2016). Photoreception and vision in the ultraviolet. J. Exp. Biol. *219*, 2790–2801.
66. Peng, Y.-R., Shekhar, K., Yan, W., Herrmann, D., Sappington, A., Bryman, G.S., van Zyl, T., Do, M.T.H., Regev, A., and Sanes, J.R. (2019). Molecular classification and comparative taxonomics of foveal and peripheral cells in primate retina. Cell *176*, 1222–1237.e22.
67. Dacey, D.M. (2000). Parallel pathways for spectral coding in primate retina. Annu. Rev. Neurosci. *23*, 743–775.
68. Jacoby, J., and Schwartz, G.W. (2017). Three small-receptive-field ganglion cells in the mouse retina are distinctly tuned to size, speed, and object motion. J. Neurosci. *37*, 610–625.
69. Novales Flamarique, I. (2016). Diminished foraging performance of a mutant zebrafish with reduced population of ultraviolet cones. Proc. Biol. Sci. *283*, 20160058.

70. Kwan, K.M., Fujimoto, E., Grabher, C., Mangum, B.D., Hardy, M.E., Campbell, D.S., Parant, J.M., Yost, H.J., Kanki, J.P., and Chien, C.-B. (2007). The Tol2kit: a multisite gateway-based construction kit for Tol2 transposon transgenesis constructs. Dev. Dyn. *236*, 3088–3099.

71. Suli, A., Guler, A.D., Raible, D.W., and Kimelman, D. (2014). A targeted gene expression system using the tryptophan repressor in zebrafish shows no silencing in subsequent generations. Development *141*, 1167–1174.

72. Karlsson, J., von Hofsten, J., and Olsson, P.E. (2001). Generating transparent zebrafish: a refined method to improve detection of gene expression during embryonic development. Mar. Biotechnol. (NY) *3*, 522–527.

73. White, R.M., Sessa, A., Burke, C., Bowman, T., LeBlanc, J., Ceol, C., Bourque, C., Dovey, M., Goessling, W., Burns, C.E., and Zon, L.I. (2008). Transparent adult zebrafish as a tool for in vivo transplantation analysis. Cell Stem Cell *2*, 183–189.

74. Thisse, C., Thisse, B., Schilling, T.F., and Postlethwait, J.H. (1993). Structure of the zebrafish snail1 gene and its expression in wild-type, spadetail and no tail mutant embryos. Development *119*, 1203–1215.

75. Ren, J.Q., McCarthy, W.R., Zhang, H., Adolph, A.R., and Li, L. (2002). Behavioral visual responses of wild-type and hypopigmented zebrafish. Vision Res. *42*, 293–299.

76. Dreosti, E., Odermatt, B., Dorostkar, M.M., and Lagnado, L. (2009). A genetically encoded reporter of synaptic activity in vivo. Nat. Methods *6*, 883–889.

77. Dana, H., Mohar, B., Sun, Y., Narayan, S., Gordus, A., Hasseman, J.P., Tsegaye, G., Holt, G.T., Hu, A., Walpita, D., et al. (2016). Sensitive red protein calcium indicators for imaging neural activity. eLife *5*, e12727.

78. Zimmermann, M.J.Y., Chagas, A.M., Bartel, P., Pop, S., Prieto Godino, L.L., and Baden, T. (2020). LED Zappelin': an open source LED controller for arbitrary spectrum visual stimulation and optogenetics during 2-photon imaging. bioRxiv. https://doi.org/10.1101/2020.03.10.986000.

79. Ran, Y., Huang, Z., Baden, T., Schubert, T., Baayen, H., Berens, P., Franke, K., and Euler, T. (2020). Type-specific dendritic integration in mouse retinal ganglion cells. Nat. Commun. *11*, 2101.

80. Berens, P. (2009). CircStat : a MATLAB toolbox for circular statistics. J. Stat. Softw. *31*, 1–21.

CellPress
OPEN ACCESS

STAR★METHODS

KEY RESOURCES TABLE

REAGENT or RESOURCE	SOURCE	IDENTIFIER
Antibodies		
Chicken anti-GFP	AbCam	13970; RRID: AB_300798
Rabbit anti-GABA	Sigma	A2052; RRID: AB_477652
Donkey anti-rabbit IgG CF568 conjugate	Sigma	SAB4600076
Donkey anti-chicken IgG CF488A conjugate	Sigma	SAB4600031; RRID: AB_2721061
Chemicals, Peptides, and Recombinant Proteins		
Paraformaldehyde	Agar Scientific	R1026
Triton X-100	Sigma	X100
Hoechst 33342	Invitrogen	H21492
BODIPY	Invitrogen	C34556
1-phenyl-2-thiourea	Sigma	P7629
α-bungarotoxin	Tocris	2133
Agarose low melting	FisherScientific	BP1360-100
DiD	Invitrogen	D307
VectaShield	Vector	H-1000
Sodium borohydride	Sigma	452882
Tween-20	Sigma	P9416
Deposited Data		
All population kernel and chirp data as well as all anatomical clustering data.	This paper, DataDryad	https://datadryad.org/stash/dataset/doi:10.5061/dryad.7sqv9s4pm
Experimental Models: Organisms/Strains		
Danio rerio (zebrafish): *Tg(Ptf1a:dsRed), Tg(Islet2b:nls-trpR, tUAS:MGCamp6f), Tg(Islet2b:nls-trpR, tUAS:SyjRGeco1a), Tg(tUAS:paGFP)*	[37]	N/A
Recombinant DNA		
pTol2CG2-tUAS-SyjRGeco1a	This paper	N/A
pME-SyjRGeco1a	This paper	N/A
pTol2BH-tUAS-paGFP	This paper	N/A
pME-paGFP	This paper	N/A
pDestTol2CG2	[70]	N/A
p5E-tUAS	[71]	N/A
p3E-pA	[70]	N/A
pTol2pA-islet2b-nlsTrpR	[37]	N/A
pTol2BH-tUAS-MGCamp6f	[37]	N/A
Software and Algorithms		
MATLAB code used for morphological and functional clustering including the data used for clustering	This paper, DataDryad	https://datadryad.org/stash/dataset/doi:10.5061/dryad.7sqv9s4pm
Igor Pro 6	Wavemetrics	N/A
ImageJ	N/A	https://imagej.nih.gov/ij/

RESOURCE AVAILABILITY

Lead Contact

Further information and requests for resources and reagents should be directed to and will be fulfilled by the lead contact, Tom Baden (t.baden@sussex.ac.uk).

Materials Availability

Plasmids pTol2CG2-tUAS-SyjRGeco1a, pTol2BH-tUAS-paGFP, pME-SyjRGeco1a, pME-paGFP, and transgenic lines *Tg(Islet2b:nls-trpR, tUAS:SyjRGeco1a)* and *Tg(tUAS:paGFP),* generated in this study, are available upon request to the lead contact.

Data and Code Availability

Pre-processed functional data as well as single-RGC morphological data, associated summary statistics, cluster allocations (where applicable) and basic analysis and clustering scripts written in MATLAB and can be accessed from DataDryad via the relevant links on http://www.retinal-functomics.net and as linked in the Key Resources Table.

EXPERIMENTAL MODEL AND SUBJECT DETAILS

Animals

All procedures were performed in accordance with the UK Animals (Scientific Procedures) act 1968 and approved by the animal welfare committee of the University of Sussex. Adult animals were housed under a standard 14/10 light/dark cycle and fed 3 times daily. Larvae (~3 mm body length) were grown in E2 solution (1.5 M NaCl, 50 mM KCl, 100 mM $MgSO_4$, 15 mM KH_2PO_4, 5 mM Na_2HPO_4) or fish water and treated with 200 μM 1-phenyl-2-thiourea (Sigma, P7629) from 12 hours post fertilization (*hpf*) to prevent melanogenesis [72]. For 2-photon in-vivo imaging, zebrafish larvae were immobilized in 2% low melting point agarose (Fisher Scientific, BP1360-100), placed on a glass coverslip and submerged in fish water. Eye movements were prevented by injection of α-bungarotoxin (1 nL of 2 mg/ml; Tocris, Cat: 2133) into the ocular muscles behind the eye.

For all experiments, we used 6-8 *dpf* zebrafish (*Danio rerio*) larvae (~3 mm body-length). The following previously published transgenic lines were used: *Tg(Ptf1a:dsRed)* [43], *Tg(Islet2b:nls-trpR, tUAS:MGCamp6f)* [37] as well as Casper [73], nacre [74] and roy [75]. In addition, two transgenic lines *Tg(Islet2b:nls-trpR, tUAS:SyjRGeco1a)* and *Tg(tUAS:paGFP)* were generated by injecting plasmid solution into one-cell stage embryos. Plasmid solutions used are; a mixture of pTol2pA-islet2b-nlsTrpR [37] and pTol2CG2-tUAS-SyjRGeco1a for the *Tg(islet2b:nls-trpR, tUAS:SyjRGeco1a)* line and pTol2BH-tUAS-paGFP for the *Tg(tUAS:paGFP)* line. Expression of paGFP was then obtained by crossing these two lines. With this combination, RGCs also express SyjRGeco1a, which was not used in this study (and which did not interfere with the green channel used for paGFP detection.

Plasmids were constructed by means of a attL/attR (LR)-reaction using destination and entry plasmids as follows: for pTol2CG2-tUAS-SyjRGeco1a; pDestTol2CG2 [70], p5E-tUAS [71], pME-SyjRGeco1a, p3E-pA [70], for pTol2BH-tUAS-paGFP; pDestTol2BH [27], p5E-tUAS, pME-paGFP, p3E-pA. pME-SyjRGeco1a was constructed by inserting PCR amplified zebrafish synaptophysin without stop codon [76] followed by PCR amplified jRGeco1a fragment [77] into pME plasmid. Similarly, pME-paGFP was constructed by inserting PCR amplified paGFP fragment into pME plasmid.

For transient expression of mGCaMP6f under Islet2b we injected a mixture of pTol2pA-islet2b-nlsTrpR and pTol2BH-tUAS-MGCamp6f plasmids [37] solution into one-cell stage eggs. Positive embryos were screened under 2-photon.

METHOD DETAILS

Tissue preparation, immunolabeling, and imaging

For immunohistochemistry, larvae were euthanized by tricaine overdose (800 mg/l) and fixed in 4% paraformaldehyde in phosphate-buffered saline (PBS) for 30 minutes at room temperature before being washed in calcium-negative PBS. Retinae were then incubated in permeabilization/blocking buffer (PBS with 0.5% Triton X-100 and 5% normal donkey serum) at 4°C for 24 hours, and thereafter transferred to the appropriate labeling solution. For nuclear labeling, tissue was incubated at 4°C in blocking solution with Hoechst 33342 nuclear dye (Invitrogen, H21492, 1:2000) for 24 hours. For membrane staining, tissue was incubated at 4°C in blocking solution with BODIPY membrane dye (Invitrogen, C34556, 1:1000) for 24 hours. For immunostaining, tissue was incubated at 4°C for 72 hours in primary antibody solution (chicken anti-GFP (AbCam, 13970, 1:500), rabbit anti-cox iv (AbCam, 16056, 1:500), diluted in permeabilization/blocking solution). Samples were rinsed three times in PBS with 0.5% Triton X-100, then transferred to secondary antibody solution (donkey anti-chicken IgG CF488A conjugate (Sigma, SAB4600031, 1:500), donkey anti-rabbit IgG CF568 conjugate (Sigma, SAB4600076, 1:500)), diluted in permeabilization/blocking solution and incubated at 4°C for 24 hours. Finally, samples were rinsed three times in PBS with 0.5% Triton X-100 before being mounted in mounting media (VectaShield, Vector, H-1000) for confocal imaging.

GABA immunostaining was performed using rabbit anti-GABA (Sigma, A2052, 1:500) according to the protocol described in [43]. Briefly, whole retinas were fixed in 2% PFA /2% glutaraldehyde for 24 hours at 4°C, rinsed in PBS, treated with 0.1% sodium borohydride ($NaBH_4$) in 0.2% Triton X-100 in PBS for 10 minutes at room temperature, and rinsed again to remove excess $NaBH_4$. For immunolabeling, all steps are as described above, with the following exceptions: blocking buffer consisted of 10% normal donkey serum, 0.1% Tween-20, and 0.5% Triton X-100 in PBS; primary and secondary antibodies were also diluted in this blocking buffer.

Confocal stacks and individual images were taken on Leica TCS SP8 using 40x water-immersion objective at xy resolution of 2,048x2,048 pixels (pixel width: 0.162 μm). Voxel depth of stacks was taken at z-step 0.3-0.5 μm. Contrast and brightness were adjusted in Fiji (NIH).

Cell density mapping

The 3D positions of all GCL somata (stained with Hoecht 3342), as well as dAC and AC somata (*tg(Ptf1a:dsRed)*, and MG *tg(GFAP:GFP),* immunolabeled against GFP) were semi-automatically detected in Fiji from confocal image stacks of intact, whole eyes. These positions were then projected into a local-distance preserving 2D map as shown previously [20] using custom-written scripts in Igor Pro 6.37 (Wavemetrics). The density map of RGC somata was computed by subtracting the density map of dACs from that of GCL cells. Similarly, the density map of ACs was computed by summing the density maps of dACs and ACs from the inner nuclear layer. From here, RGC maps were also mapped into a sinusoidal projection of visual space [27].

Axonal tracing

The lipophilic tracer dye DiO (Invitrogen, D307) was used to trace RGC axons from the retina to their arborization fields in the pretectum and tectum. 1 mg/mL stock solution was prepared in dimethylformamide and stored at −20°C. For injection into *Tg(Islet2b:nls-trpR, tUAS:MGCamp6f)* retinas, the lenses of whole fixed larvae were removed and a sufficient amount of tracer dye injected into one of either the left or the right eye so as to completely cover the exposed surface of the GCL. Tissue was then incubated at 37°C for 3 days to allow the dye time to diffuse all the way up RGC axons to their terminals in the midbrain.

Photoactivation

Prior to photoconversion, 6-8 *dpf* Islet2b:PA-GFP larvae were injected with BODIPY membrane dye (1 nL of 1 mg/mL; Sigma, D3821) into the space behind the right eye and underlying skin to demarcate retinal anatomy and facilitate subsequent targeting. Larvae were left for 10-20 minutes at 25°C to allow the dye to diffuse into the retina. After 20 minutes, the IPL was uniformly stained, and the individual somata of GCL neurons showed nuclear exclusions which were used for subsequent targeting.

Cells were photoconverted under the same 2-photon microscope as used for functional imaging (below). In each animal, we randomly photoconverted 2-5 cells per eye in the nasal retina and/or strike zone, with a minimum spacing of 30 μm between them. For photoactivation, the femtosecond laser was tuned to 760 nm and focused onto one single soma at a time for up to ∼2 minutes. After a typically > 40 minutes cells were visualized under 2-photon (927 nm) and imaged in a 512x512 pixel (1 μm z-steps) stack which encompassed each cell’s soma, axon initial segment, and the entirety of the dendritic structure. Throughout, the BODIPY signal was included as an anatomical reference.

Two-photon functional imaging and stimulation parameters

For all *in vivo* imaging experiments, we used a MOM-type two-photon microscope (designed by W. Denk, MPI, Martinsried [38]; purchased through Sutter Instruments/Science Projects) equipped with the following: a mode-locked Ti:Sapphire laser (Chameleon Vision-S, Coherent) tuned to 927 nm for imaging GFP and 960 nm for imaging mCherry/BODIPY in combination with GFP; two fluorescent detection channels for GFP (F48x573, AHF/Chroma) and mCherry/BODIPY (F39x628, AHF/Chroma), and; a water-immersion objective (W Plan-Apochromat 20x/1,0 DIC M27, Zeiss). For image acquisition, we used custom-written software (ScanM, by M. Mueller, MPI, Martinsried and T Euler, CIN, Tübingen) running under Igor Pro 6.37 (Wavemetrics). Structural data was recorded at 512x512 pixels, while functional data was recorded at 64x32 pixel resolution (15.6 Hz, 2 ms line speed). For each functional scan, we first defined a curvature of the imaged IPL segment based on a structural scan, and thereafter “bent” the scan plane accordingly (“banana scan”). This ensured that the imaging laser spent a majority of time sampling from the curved IPL and INL, rather than adjacent dead space. The banana-scan function was custom-written under ScanM.

For light stimulation, we focused a custom-built stimulator through the objective, fitted with band-pass-filtered light-emitting diodes (LEDs) (‘red’ 588nm, B5B-434-TY, 13.5 cd, 8°; ‘green’ 477 nm, RLS-5B475-S, 3-4cd, 15°, 20 mA; ‘blue’ 415 nm, VL415-5-15, 10-16 mW, 15°, 20 mA; ‘ultraviolet’ 365 nm, LED365-06Z, 5.5 mW, 4°, 20 mA; Roithner, Germany). LEDs were filtered and combined using FF01-370/36, T450/pxr, ET420/40 m, T400LP, ET480/40x, H560LPXR (AHF/Chroma). The final spectra approximated the peak spectral sensitivity of zebrafish R-, G-, B-, and UV-opsins, respectively, while avoiding the microscope’s two detection bands for GFP and mCherry/BODIPY. To prevent interference of the stimulation light with the optical recording, LEDs were synchronized with the scan retrace at 500Hz (2 ms line duration) using a microcontroller and custom scripts. Further information on the stimulator, including all files and detailed build instructions can be found at [78].

Stimulator intensity was calibrated (in photons per second per cone) such that each LED would stimulate its respective zebrafish cone type with a number of photons adjusted to follow the relative power distribution of the four wavelength peaks of daytime light in the zebrafish natural habitat [20, 23] to yield ‘natural white’: red, “100%” (34x10^5 photons /s /cone); green, “50%” (18 x10^5 photons /s /cone); blue, “13%” (4.7 x10^5 photons /s /cone); ultraviolet, “6%” (2.1x10^5 photons /s /cone). We did not compensate for cross-activation of other cones. Owing to 2-photon excitation of photopigments, an additional constant background illumination of ∼10^4 R* was present throughout [38, 39]. For all experiments, larvae were kept at constant illumination for at least 2 s after the laser scanning started before light stimuli were presented. Two types of full-field stimuli were used: a binary dense “natural spectrum” white noise, in which the four LEDs were flickered independently in a known random binary sequence at 6.4 Hz for 258 s, and a natural-white chirp stimulus [2] where all four LEDs were driven together. To prevent interference of the stimulation light with the optical recording, LEDs were synchronized to the scanner’s retrace [39].

QUANTIFICATION AND STATISTICAL ANALYSIS

No statistical methods were used to predetermine sample size.

Data analysis

Data analysis was performed using IGOR Pro 6.3 (Wavemetrics), Fiji (NIH) and MATLAB R2018b (Mathworks).

ROI placements and quality criterion

ROIs were automatically placed using local image correlation based on established protocols – for details see [41]. To allocate ROIs to dendritic and somatic datasets a boundary between the GCL and IPL was drawn by hand in each scan - all ROIs with a center of mass above the boundary were considered as dendritic, and all ROIs below were considered as somatic. Since the lower part of the IPL tends to be dominated by On-circuits, it is possible that a small number of On-dendrites were incorrectly classed as somata which may go part-way to explaining the generally stronger On-bias among somatic compared to dendritic ROIs (e.g., Figure 2A). Moreover, due to the ring-like nature of mGCaMP6f expression profiles in somata when optically sectioned, it was possible that two ROIs could be inadvertently placed on different halves of the same soma. However, since whether or not a soma was split in this way was likely non-systematic over functional types, we did not attempt to correct for this possibility. Only ROIs where at least one of the four spectral kernels' peak-to-peak amplitudes exceeded a minimum of ten standard deviations were kept for further analysis (n = 2,414/2,851 dendritic ROIs, 84.7%; 411/796 somatic ROIs, 51.6%). Equally, all individual color kernels that did not exceed 10 SDs were discarded (i.e set to NaN).

A note on ROI segmentation and identity

We used 2-photon imaging of Islet2b:mGCaMP6f signals in the eye's GCL and IPL to functionally survey RGC functions in larval zebrafish. While this approach likely provides for a useful approximation of what the zebrafish's eye tells the zebrafish's brain, two main caveats must be considered. First, while Islet2b is an effective and popular marker for zebrafish RGCs it is neither exclusive to RGCs nor inclusive of all RGCs. In our Islet2b:mGCaMP6f line, immunostaining against GFP and GABA revealed that some dACs also express GCaMP6f (Figure S1B), indicating that our dataset contains a minority of signals from dACs. In addition, small numbers of INL somata are labeled, indicating that also a minority of ACs contribute to our dendritic signals (AC somata are not included since these are easily discarded based on location). Conversely, not all axonal arborisation fields (AFs) in the brain, as revealed after DiO injection into the eye, were also strongly innervated by mGCaMP6f expressing RGCs (Figure S1C), suggesting that a subset of RGC types may be absent in our dataset. Finally, also a small fraction of central neurons were labeled as evident from their soma locations near the (pre)tectal neuropils. Second, population imaging of RGC dendrites in the eye is potentially fraught with many of the same problems that are associated with delineating their axonal signals in the brain [17]. Specifically, the high density and overlap of dendritic processes across the IPL means that it is impossible to tell if groups of dendritic ROIs belong to the same RGC (Figures 1D and 1E). Nevertheless, functional dendritic data is indicative of the local computations that occur within RGC dendrites as they integrate signals from BCs and ACs in different layers of the IPL and in different positions of the eye [79]. Further, our single cell data (Figures S2A–S2E) suggests that dendritic signals in population recordings are probably also a reasonable proxy for somatic signals, with the added benefit that their signal-to-noise was generally higher (e.g., Figure 2A). To what extent the indicated close similarity of dendritic and somatic signals in zebrafish RGCs applies across all RGC types, and to what extent this can be linked to their generally small absolute size (e.g., compared to RGCs in larger eyes), will be important to address in the future.

The somata of RGCs in the GCL could generally be reliably segmented in population recordings. In view of their proximity to the axon hillock, data from RGC somata may serve as a useful indication of the signal sent from the eye to the brain. Nevertheless, addressing how exactly somatic calcium signals are linked to spikes sent down the optic nerve will be important in the future. This may then also go partway to explaining the marked reduction in Off-responses in somatic data compared to dendrites (Figures 2A, 2B, 3A, and 5B), and more broadly to drive our understanding of how this tiny animal's eye communicates with its brain.

Kernel polarity

The use of a fluorescence-response-triggered average stimulus (here: 'kernel') as a shorthand for a neuron's stimulus-response properties, while potentially powerful (e.g., [20, 41]), ought to be considered with some caution. For example, determining a binary value for a kernel's polarity (On or Off) can be conflicted with the fact that a neuron might exhibit both On and Off response aspects. Moreover, different possible measures of On or Off dominance in a kernel can generate different classification biases. Here, we defined On and Off based on a measure of a kernel's dominant trajectory in time. For this, we determined the position in time of each kernel's maximum and minimum. If the maximum preceded the minimum, the kernel was classified as Off, while vice versa if the minimum preceded the maximum, the kernel was defined as On. Examples On and Off kernels classified in this way can for example be seen in Figure 3B (*cf.* Figure 3A central horizontal column for a lookup of how each kernel was classified).

Digitizing photoactivated cells

Dendritic swellings (together taken as a proxy for the overall stratification profile of the dendritic tree) in photoconverted GCL cells were detected using Fiji. For this, the GFP channel was smoothed and thresholded to create a binary mask removing background fluorescence. Any remaining neurites that clearly did not belong to the most strongly labeled cell were removed by hand. Next,

the soma and any dendritic swellings were automatically detected using 3D Objects Counter plugin in Fiji. 3D positions of all detected objects were then normalized relative to the boundaries of the IPL, as determined from the BODIPY channel. This generated an IPL-aligned 3D 'dot-cloud' for each RGC, which was then used as the input for a custom clustering algorithm. We also projected each dot-cloud into *en-face* and side-view density maps for visualization. Note that sideview projections shown in Figure S6G are laterally compressed five-fold to highlight differences in stratification depths across the IPL.

Quantifying dendritic tilt

As noted in 'Morphology Clustering' (below), morphological data consists of sets of points in three-dimensional Cartesian coordinates (x,y,z) describing the location of the soma and the dendritic architecture for each RGC. The coordinate axes are orientated such that the y axis is perpendicular to the plane of the retina, pointing outward, away from the center of the eye, while the x and z axes are tangential to the plane of the retina. We translated the coordinate system for each cell such that its soma lies at the origin. We then calculated the center of mass (CoM) of the point cloud representing the dendritic tree of each cell (i.e., excluding the soma), computed as the mean of the points' x, y and z positions. We then transformed to a spherical polar coordinate system, (r,θ,φ), with the origin centered at the soma, where $r > 0$ (μm) is the distance of the dendritic CoM from the soma, the polar angle $0 \leq \theta \leq \pi$ (rad), characterizes the dendritic tilt strength (i.e., the angle subtended by the dendritic CoM from the y axis, where $\theta = 0$ corresponds to no tilt and $\theta = \pi/2$ occurs when the dendritic CoM has the same IPL/GCL depth as the soma) and the azimuthal angle, $0 \leq \varphi\ 2\pi$(rad), characterizes the dendritic tilt direction. It should be noted that the relationship between our Cartesian and spherical polar coordinate systems is different from that which is standard in that we have swapped the y and z axes. Thus, the polar angle is subtended from the y axis, rather than from the z-axis as is usual.

We tested whether the distributions of the position of the dendritic CoM relative to the soma in each of the r, θ and φ dimensions for SZ and nasal RGCs are from the same (continuous) distribution using the two-sample Kolmogorov-Smirnov test. This was implemented using the MATLAB routine kstest2 for r and θ, and using the circ_kuipertest routine from the CircStat toolbox [80] for φ, since this variable is (2π-)periodic. In comparing SZ and nasal RGCs, the dendritic CoM positions, r, are predicted to be from different distributions ($p = 0.0209$, 3 s.f.); the dendritic tilt strengths, θ, are predicted to be from the same distribution ($p = 0.894$, 3 s.f.); and the dendritic tilt angles, φ, are predicted to be from different distributions ($p = 0.001$).

Morphology Clustering

The morphological data consists of sets of points in three-dimensional Cartesian coordinates (x,y,z) describing the dendritic architecture for each of 131 RGCs, 67 from the nasal (N) region and 64 from the strike zone (SZ) region. The coordinate axes are orientated such that the y axis is perpendicular to the plane of the retina, spanning the width of the IPL, while the x and z axes are tangential to the plane of the retina. The coordinates in the y-dimension are scaled so as to lie in the interval [0,10] for any processes within the IPL, and > 10 or < 0 for INL and GCL processes (where applicable), respectively. The position of the soma, which always lay in the GCL, was not used for clustering.

Three summary statistics, each of which capture some aspect of the dendritic architecture, were defined for use in clustering: i) y_span: the width of the dendritic tree in the y-direction; ii) y_mean: the mean position of the points in the dendritic tree in the y-direction; and iii) num_pts: the number of points in the dendritic tree. While we experimented with other summary statistics, these three were found to be sufficient to differentiate the RGCs into their basic morphological groups.

We also defined one further summary statistic: iv) xz_area: the area spanned by the dendritic tree in the xz-plane, calculated as the convex hull using the MATLAB routine convhull. This statistic was not used for clustering since the information contained in xz_area is largely captured between y_span and num_pts. While not required for clustering, this summary statistic nonetheless captures important characteristics of the dendritic morphology and hence is represented in the results section alongside y_span, y_mean and num_pts.

Each of the summary statistics was standardized by subtracting the mean and dividing by the standard deviation. In this way, we ensured that each of the summary statistics was equally weighted by the clustering algorithm.

Clustering was performed in two stages, using agglomerative hierarchical clustering in both cases. The first stage of clustering used all three summary statistics (y_span, y_mean and num_pts), splitting the data into 18 clusters. Two of the resulting clusters were large and contained a variety of morphologies as discerned from visual inspection. These clusters were split further via a second round of clustering, using just the y_span summary statistic. The first cluster was split into 6 subclusters and the second into 3 subclusters, resulting in a total of 25 clusters, where the 13 clusters containing a minimum of 4 members were included for presentation.

Hierarchical clustering was performed using the MATLAB routines pdist, linkage and cluster. The function pdist calculates the distances between each RGC in (y_span,y_mean,num_pts)-space, while the function linkage operates on the output of the pdist routine to encode an agglomerative hierarchical cluster tree. There are a number of options for defining the distances between RGCs for pdist and the distances between clusters for linkage. We used the 'city block' distance metric for pdist and the 'average' distance metric for linkage as, in general, these were found to result in a larger cophenetic correlation coefficient (CCC) than any other combination of distance metrics. The CCC is a measure of the fidelity with which the cluster tree represents the dissimilarities between observations. It was calculated using the MATLAB routine cophenet and takes values between [-1,1], where values closer to positive unity represent a more faithful clustering. In the results presented here, the first stage of clustering had a CCC of 0.77 (2 d.p.), while the two subclusterings in the second stage had CCCs of 0.77 (2 d.p.) and 0.83 (2 d.p.).

Lastly, RGCs were assigned to clusters using the MATLAB routine cluster. The number of clusters was determined by specifying a cutoff distance which was chosen following visual inspection of the cluster tree dendrogram so as to respect a natural division in the data.

Functional data pre-processing and receptive field mapping

Regions of interest (ROIs), corresponding to dendritic or somatic segments of RGCs were defined automatically as shown previously based on local image correlation over time [41]. Next, the Ca^{2+} traces for each ROI were extracted and de-trended by high-pass filtering above ~0.1 Hz and followed by z-normalization based on the time interval 1-6 s at the beginning of recordings using custom-written routines under IGOR Pro. A stimulus time marker embedded in the recording data served to align the Ca^{2+} traces relative to the visual stimulus with a temporal precision of 1 ms. Responses to the chirp stimulus were up-sampled to 1 KHz and averaged over 3-6 trials. For data from tetrachromatic noise stimulation we mapped linear receptive fields of each ROI by computing the Ca^{2+} transient-triggered-average. To this end, we resampled the time-derivative of each trace to match the stimulus-alignment rate of 500 Hz and used thresholding above 0.7 standard deviations relative to the baseline noise to the times t_i at which Calcium transients occurred. We then computed the Ca^{2+} transient-triggered average stimulus, weighting each sample by the steepness of the transient:

$$\boldsymbol{F}(l,\tau) = \frac{1}{M}\sum_{i=1}^{M}\dot{c}(t_i)\boldsymbol{S}(o,t_i+\tau)$$

Here, $\boldsymbol{S}(l,t)$ is the stimulus (“LED” and “time”), τ is the time lag (ranging from approx. −1,000 to 350 ms) and M is the number of Ca^{2+} events. RFs are shown in z-scores for each LED, normalized to the first 50 ms of the time-lag. To select ROIs with a non-random temporal kernel, we used all ROIs with a standard deviation of at least ten in at least one of the four spectral kernels. The precise choice of this quality criterion does not have a major effect on the results.

Eye-IPL maps

To summarize average functions of RGC processes across different positions in the eye and across IPL depths, we computed two-dimensional “Eye-IPL” maps. For this, we divided position in the eye (-π:π radians) into eight equal bins of width π/4. Similarly, we divided the IPL into 20 bins. All soma ROIs were allocated to bin 1 independent of their depth in the GCL. while all IPL ROIs were distributed to bins 3:20 based on their relative position between the IPL boundaries. As such, bin 2 is always empty, and serves as a visual barrier between IPL and GCL. From here, the responses of ROIs within each bin were averaged. All maps were in addition smoothed using a circular π/3 binomial (Gaussian) filter along eye-position, as well as for 5% of IPL depth across the y-dimension (dendritic bins 3:20 only).

On-Off index (OOi)

For each Eye-IPL bin, an On-Off index (OOi) was computed:

$$OOi = \frac{nOn - noff}{nOn + nOff}$$

Where nOn and nOff correspond to the number of On and Off kernels in a bin, respectively. OOi ranged from 1 (all kernels On) to −1 (all kernels Off), with and OOi of zero denoting a bin where the number of On and Off kernels was equal.

Ternary response classification

Each ROI was allocated to one of 81 ternary response bins (three response states raised to the power of four spectral bands). One of three response-states was determined for each of four spectral kernels (red, green, blue, UV) belonging to the same ROI: On, Off or non-responding. All kernels with a peak-to-peak amplitude below ten standard deviations were considered non-responding, while the remainder was classified as either On or Off based on the sign of the largest transition in the kernel (upward: On, downward: Off).

Feature extraction and Clustering

Clustering was performed on four datasets, each containing the functional responses of RGCs to chirp stimuli and kernels derived from color noise stimuli: 1) pan retinal inner plexiform layer (PR-IPL) dataset (n = 2,851), sampling RGC dendritic responses at all eccentricities and across a range of depths in the IPL; 2) strike zone inner plexiform layer (SZ-IPL) dataset (n = 3,542), sampling RGCs at the SZ only and across the IPL; 3) pan retinal ganglion cell layer (PR-GCL) dataset (n = 796), sampling RGC responses at all eccentricities from the RGC somata in the GCL; and 4) strike zone ganglion cell layer (SZ-GCL) dataset (n = 1,694), sampling RGCs at the SZ only from the RGC somata. Mean responses to chirp stimuli were formatted as 2,499 time points (dt = 1 ms) while color kernels were formatted as 649 time points (dt = 2 ms, starting at t = −0.9735 s) per spectral channel (red, green, blue and UV).

For each dataset we clustered using only the kernels portion of the data since this was found to produce a cleaner clustering than when clustering chirp responses and kernels together, or chirp responses alone. ROIs with low quality kernels, determined as the maximum standard deviation across the four colors, were identified and removed from the dataset. For clustering, a kernel quality threshold of 5 was chosen, such that any ROI with a kernel quality below this threshold was eliminated from the data to be clustered.

CellPress
OPEN ACCESS

Following quality control, the datasets had the following sizes: 1) PR-IPL: n = 2,414 (84.7% of original); 2) SZ-IPL: n = 2,435 (68.8% of original); 3) PR-GCL: n = 411 (51.6% of original); 4) SZ-GCL: n = 721 (42.6% of original).

We scaled the data corresponding to each kernel color by dividing each one by the standard deviation through time and across ROIs. In this way we ensured an even weighting for each color. This is important, since the red and green kernels tended to have larger amplitudes than the blue and UV kernels.

We used principal component analysis (PCA) to reduce the dimensions of the problem prior to clustering. PCA was performed using the MATLAB routine pca (default settings). We applied PCA to the portions of a dataset corresponding to each of the kernel colors separately, retaining the minimum number of principal components necessary to explain $\geq$ 99% of the variance. The resulting four 'scores' matrices were then concatenated into a single matrix ready for clustering. The following numbers of principal components were used for each of the four datasets: 1) PR-IPL: 8 red (R) components, 8 green (G) components, 13 blue (B) components, 33 ultraviolet (UV) components (62 in total); 2) SZ-IPL: 15 R, 17 G, 25 B, 18 UV (75 in total); 3) PR-GCL: 13 R, 11 G, 24 B, 36 UV (84 in total); and 4) SZ-GCL: 20 R, 21 G, 27 B, 34 UV (102 in total).

We clustered the combined 'scores' matrix using Gaussian Mixture Model (GMM) clustering, performed using the MATLAB routine fitgmdist. We clustered the data into clusters of sizes 1,2,…,100, using i) shared-diagonal, ii) unshared-diagonal, iii) shared-full and iv) unshared-full covariance matrices, such that (100*4 =) 400 different clustering options were explored in total. For each clustering option 20 replicates were calculated (each with a different set of initial values) and the replicate with the largest loglikelihood chosen. A regularization value of 10^{-5} was chosen to ensure that the estimated covariance matrices were positive definite, while the maximum number of iterations was set at 10^4. All other fitgmdist settings were set to their default values.

In datasets PR-IPL and SZ-IPL the optimum clustering was judged to be that which minimized the Bayesian information criterion (BIC), which balances the explanatory power of the model (loglikelihood) with model complexity (number of parameters), while clusters with < 10 members were removed. In datasets PR-GCL and SZ-GCL the BIC did not give a clean clustering; therefore, we specified 20 clusters for the PR-GCL and 10 clusters for the SZ-GCL, with unshared-diagonal covariance matrices, removing clusters with < 5 members.

Using the above procedure, we obtained the following optimum number of clusters for each dataset: 1. PR-IPL: 15 clusters (2 clusters with < 10 members removed); 2. SZ-IPL: 12 clusters (1 cluster with < 10 members removed); 3. PR-GCL: 13 clusters (7 clusters with < 5 members removed); 4. SZ-GCL: 9 clusters (1 cluster with < 5 members removed). Unshared-diagonal covariance matrices gave the optimal solution in all cases.

Current Biology, Volume *30*

Supplemental Information

Zebrafish Retinal Ganglion Cells

Asymmetrically Encode Spectral

and Temporal Information across Visual Space

Mingyi Zhou, John Bear, Paul A. Roberts, Filip K. Janiak, Julie Semmelhack, Takeshi Yoshimatsu, and Tom Baden

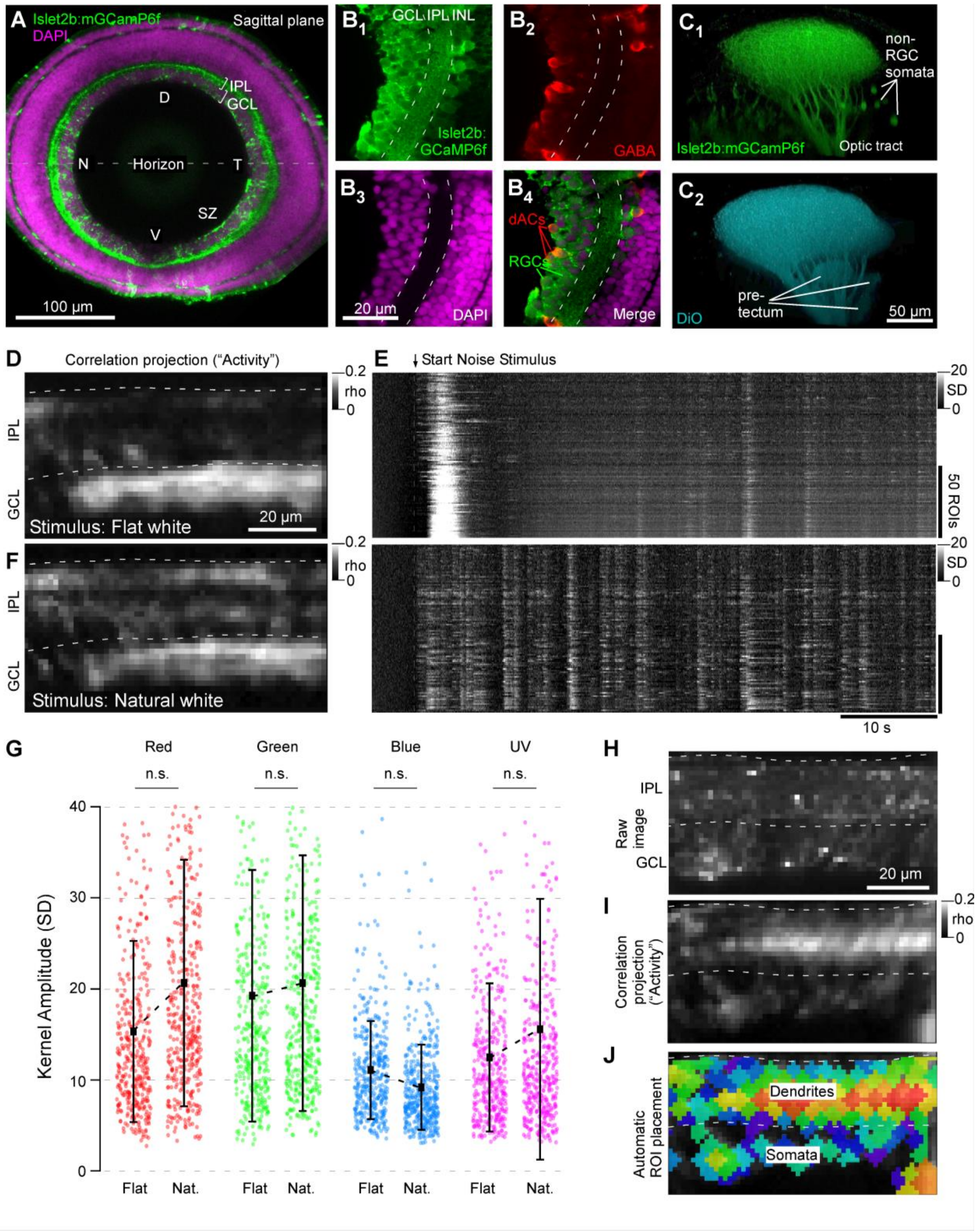

Figure S1. Islet2b expression, stimulation with "natural" white light and ROI placement. Related to Figure 1A. 7 dpf larva whole-eye sagittal plane confocal image of Islet2b:GCaMP6f expression (green) on the background of a DAPI stain, labelling all somata (magenta). D, dorsal; T, temporal; V, ventral; N, Nasal; SZ, Strike zone; INL Inner nuclear layer; GCL, Ganglion cell layer. **$B_{1\text{-}4}$**, Example higher magnification as in (A) from a second animal, with additional immunolabelling for GABA (red) to reveal GABAergic dACs and AC. Note the subset of somata showing both GABA labelling and mGCaMP6f expression (B_4). Note also the near doubling of GCL thickness across the region from the ventral retina (bottom) leading into the SZ (top). **C,** confocal projections of mGCaMP6f signal in the brain (C_1) and counter labelling by DiO injection in the eye (C_2). Though generally similar, C_1 shows expression in small numbers of brain-somata, while C_2 shows stronger labelling in pre-tectal

axonal arborisation fields. **D-F,** comparison of light-evoked activity in the same scan region in the SZ during stimulation with spectrally-flat white-noise (D, E top) and identical sequence 'natural spectrum' white noise (i.e. with green, blue and UV attenuated relative to red, cf. Figure 1B) (F, E bottom). Panels D,F show the correlation projection for the entire scan, while E top and bottom shows heatmaps of all extracted ROIs from each scan, respectively. Note the strong initial response when starting the noise-stimulus in the flat-white condition (E, top), followed by an extended period of response suppression. In contrast, ROIs during the natural-white condition responded briskly to the noise sequence without showing strong adaptation (E, bottom). Similarly, a more diverse set of scan-regions strongly responded in the natural white condition (F) compared to flat-white (D). **G**, The mean of the distributions of resultant kernel amplitudes across n=6 such scans from an identical number of animals (n=388 and 428 ROIs for the flat and natural-white condition, respectively) were indistinguishable (Wilcoxon Rank Sum Test, 2 tailed). Based on these results, we decided to use natural-white noise stimulation throughout this study. **H-J**, example scan demonstrating typical automated ROI placement. The scan was manually segmented into IPL (H, top) and GCL (H, bottom). In parallel, we computed mean correlation over time between all pairs of neighbouring pixels for the entire scan (I), and the resultant correlation-projection image was in turn used to seed and flood-fill ROIs (J). For further details and a discussion about the rationale of this approach, see [41].

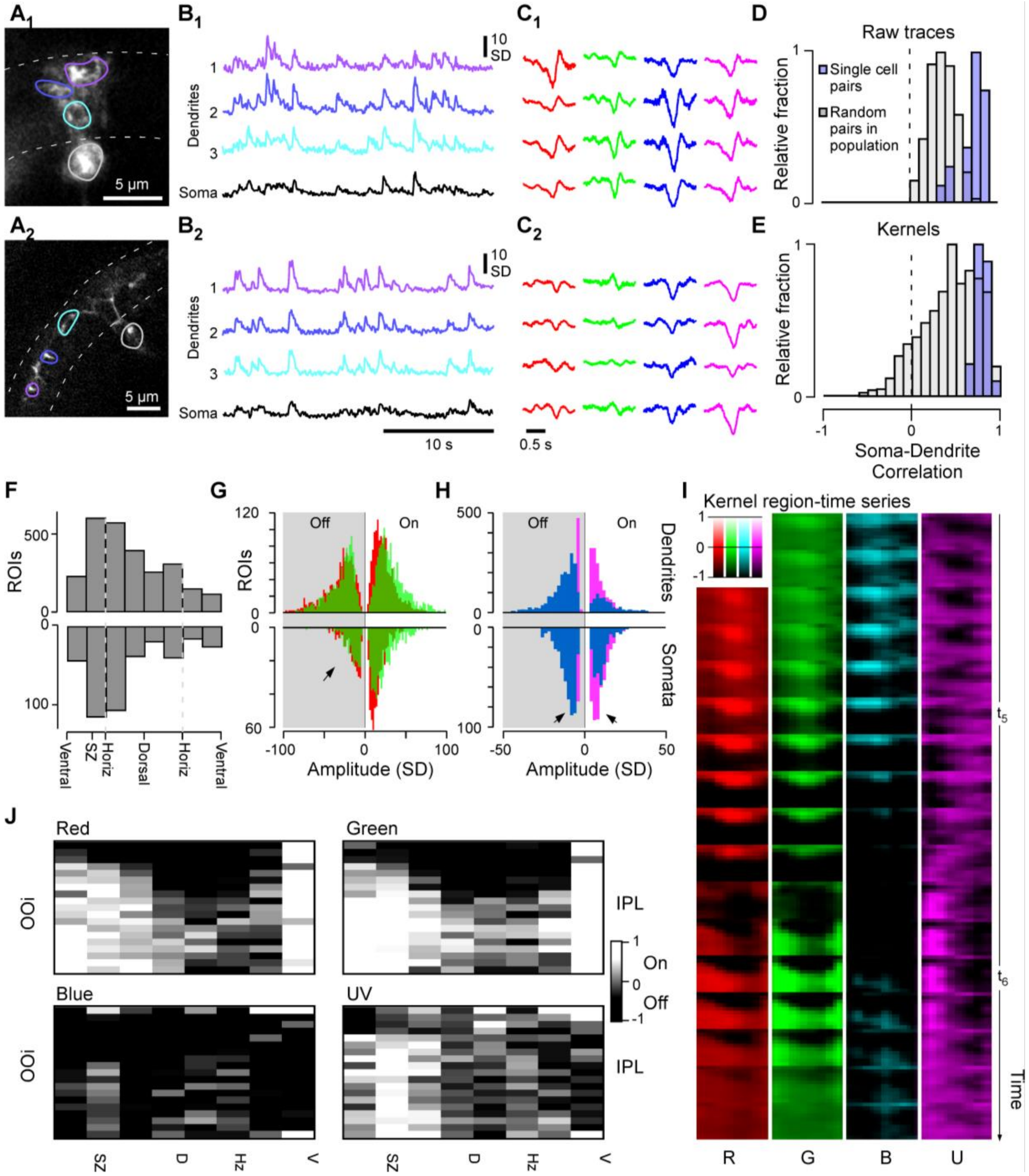

Figure S2. Linking dendritic with somatic data and eye-wide overview of RGC functions. Related to Figure 2. A-C, correlations of mGCaMP6f signals and spectral kernels from pairs of soma and dendrites belonging to the same cell, achieved by a transient expression strategy (Methods). Two RGC examples with distinct morphologies (A_1-C_1 small-field and diffuse from SZ; A_2-C_2 widefield and narrow from nasal retina) are highlighted: A, High resolution 2P scan of individual RGCs in the live eye with IPL boundaries and manually placed ROIs highlighted. B, example activity traces from each of these ROIs to naturalistic white-noise stimulation (cf. Fig S1E) and C, spectral kernels extracted from each ROI. Note that by by and large, dendritic and somatic responses (B) and kernels (C) from the same cell are very similar. **D, E**, quantification of correlations in pairs of somatic and dendritic ROIs (light purple) of raw activity traces (D) and of kernels (E). Data from n = 7 single RGCs with a total of 20 soma-dendrite pairs is shown. For comparison, the same analysis was performed for n = 400 random within-scan pairs of somatic and dendritic ROIs from n=4 nasal/dorsal population scans comprising n = 24, 16, 7, 13 somatic and n = 99, 69, 69 and 42 dendritic ROIs, respectively (light grey). In both cases, single-cell pairs were significantly more correlated than random population pairs ($p<0.001$ Wilcoxon Rank Sum test, 1 tailed), indicating that in general dendritic responses provide a useful - albeit not perfect - approximation of somatic responses. **F**, Number of dendritic (top) and

somatic (bottom, y-flipped) ROIs recorded across different positions in the eye. The relative abundance of SZ-ROIs is in line with the increased RGC numbers and thicker retinal layers [20] in this part of the eye (cf. Figure 7A). **G, H**, Distribution of dendritic (top) and somatic (bottom) On- and Off-kernels (as in Figure 2A), divided into the four wavebands, with red/green (G) and blue/UV shown together (H). Chi-Squared with Yates correction for On:Off distributions dendrites vs. somata: $p < 0.00001$ in all cases except blue, where $p = 0.0018$. **I**, Eye-IPL maps for R/G/B/U kernels (cf. Figure 2J) plotted over time (cf. Video S4) **J**, Projection of an On-Off index (OOi, Methods) in the four wavebands (as in Figure 2J) into an Eye-IPL map, with lighter and darker shades indicating an overall On- and Off- bias, respectively (see also Figure S2G,H).

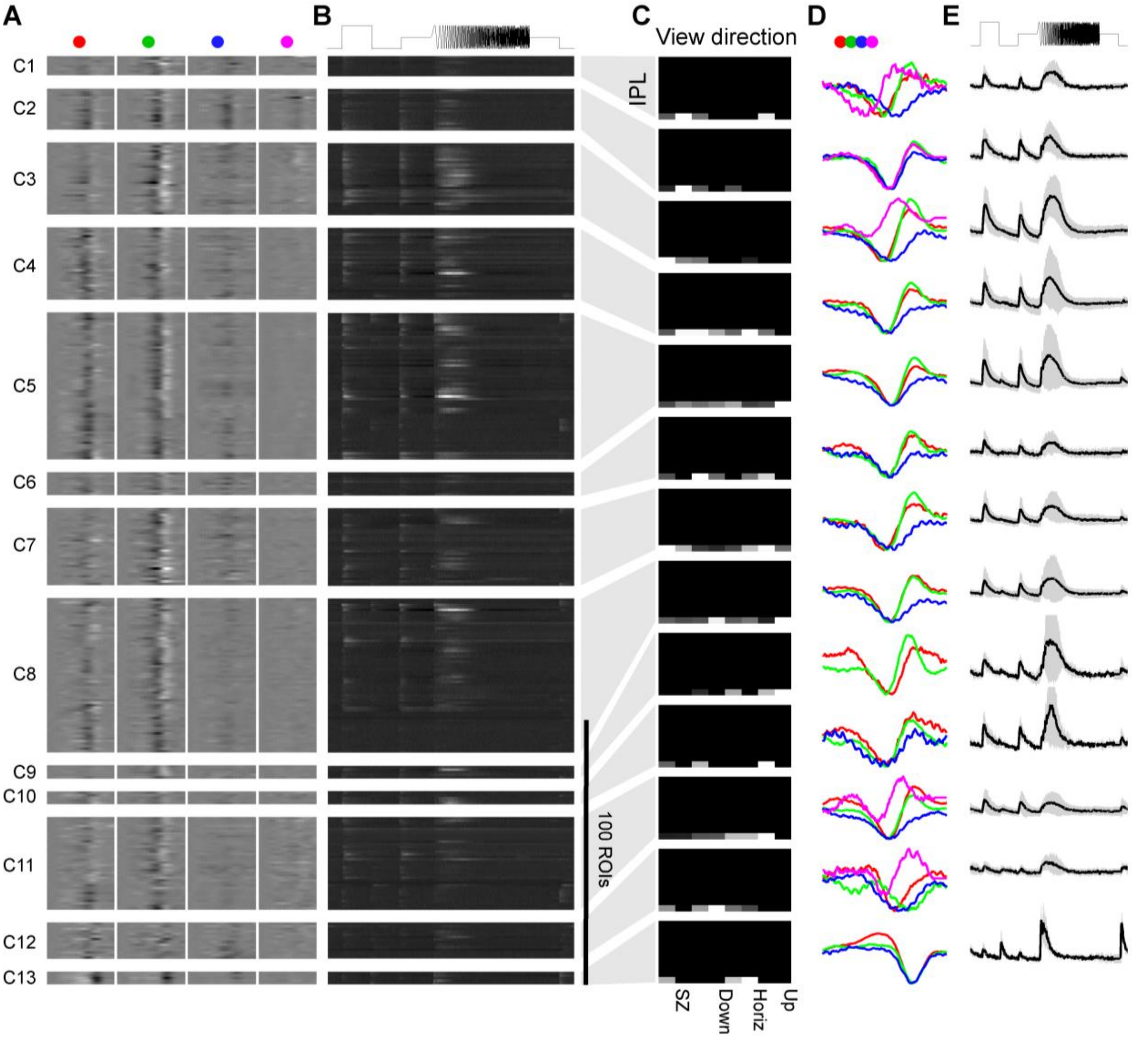

Figure S3. Functional clustering of somatic ROIs across the eye. Related to Figure 4. A-E, Somatic data from across the eye clustered based on spectral kernels, presented following the same organisation as used for dendritic data (Figure 4A-F).

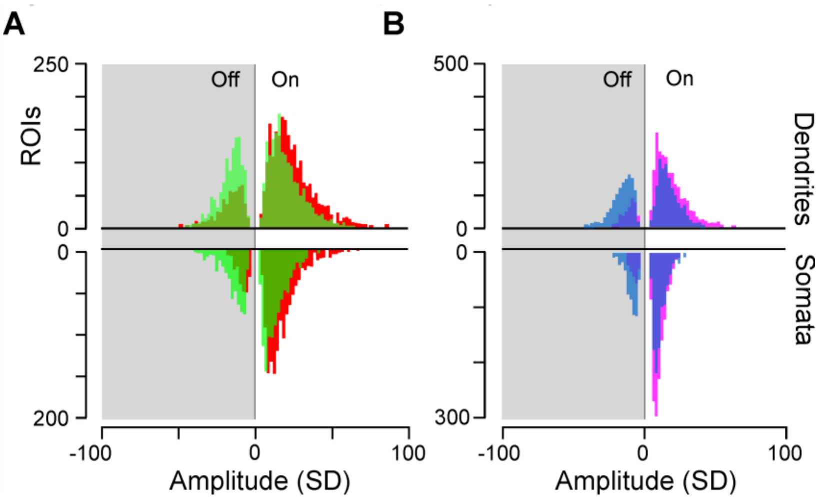

Figure S4. SZ On- and Off-responses by waveband. **Related to Figure 5. A, B**, Distribution of dendritic (top) and somatic (bottom) On- and Off-kernels (as in Figure 5B), divided into the four wavebands, with red/green (A) and blue/UV shown together (B). Dendrites n = 2,103; 1,528; 1,294; 1,864 R/G/B/U On, n = 483; 913; 984; 219 R/G/B/U Off, respectively; Somata n = 1,385; 1,034; 1,015; 1,356 R/G/B/U On, n = 236; 582; 569; 209 R/G/B/U Off, respectively. Chi-Squared with Yates correction for On:Off distributions dendrites vs. somata: p = 0.0006; 0.37; <0.00001; 0.0097 for R/G/B/U, respectively.

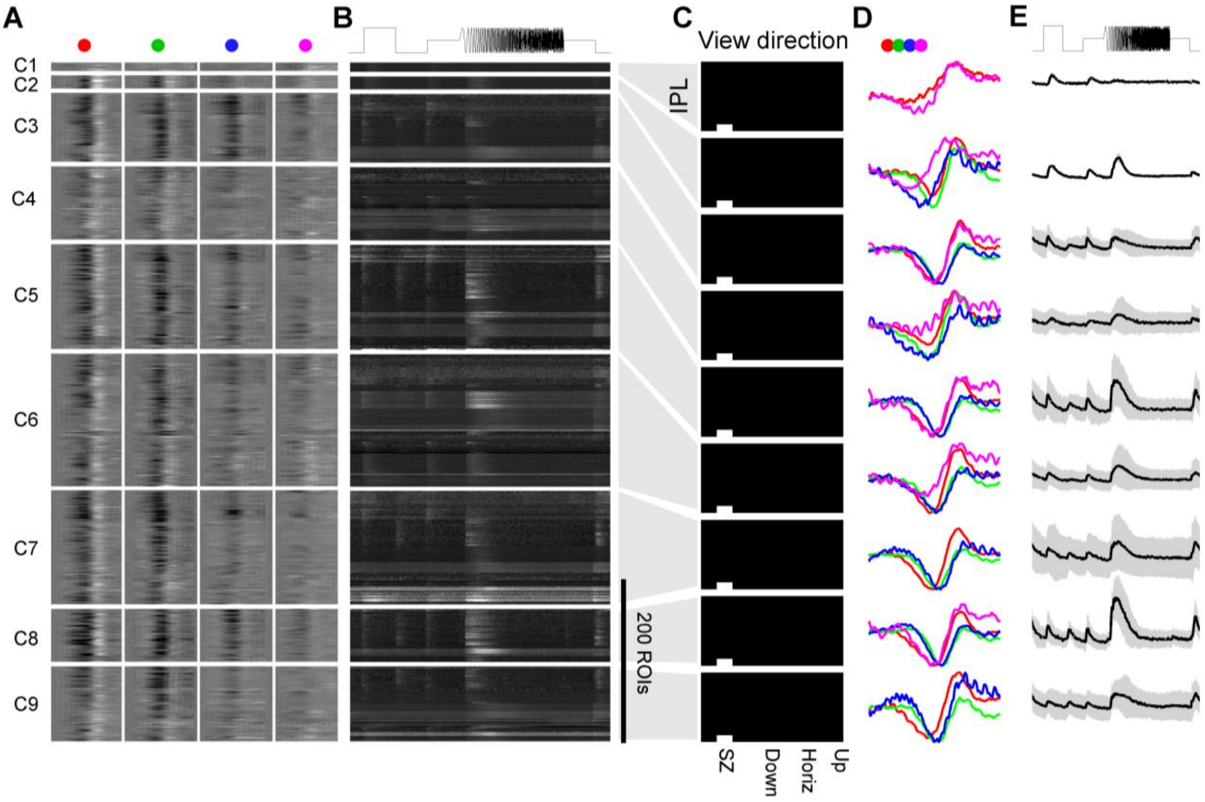

Figure S5. Functional clustering of somatic ROIs in the strike zone. **Related to Figure 6. A-E**, Somatic data from the SZ based on spectral kernels, presented following the same organisation as used for dendritic data (Figure 6A-F).

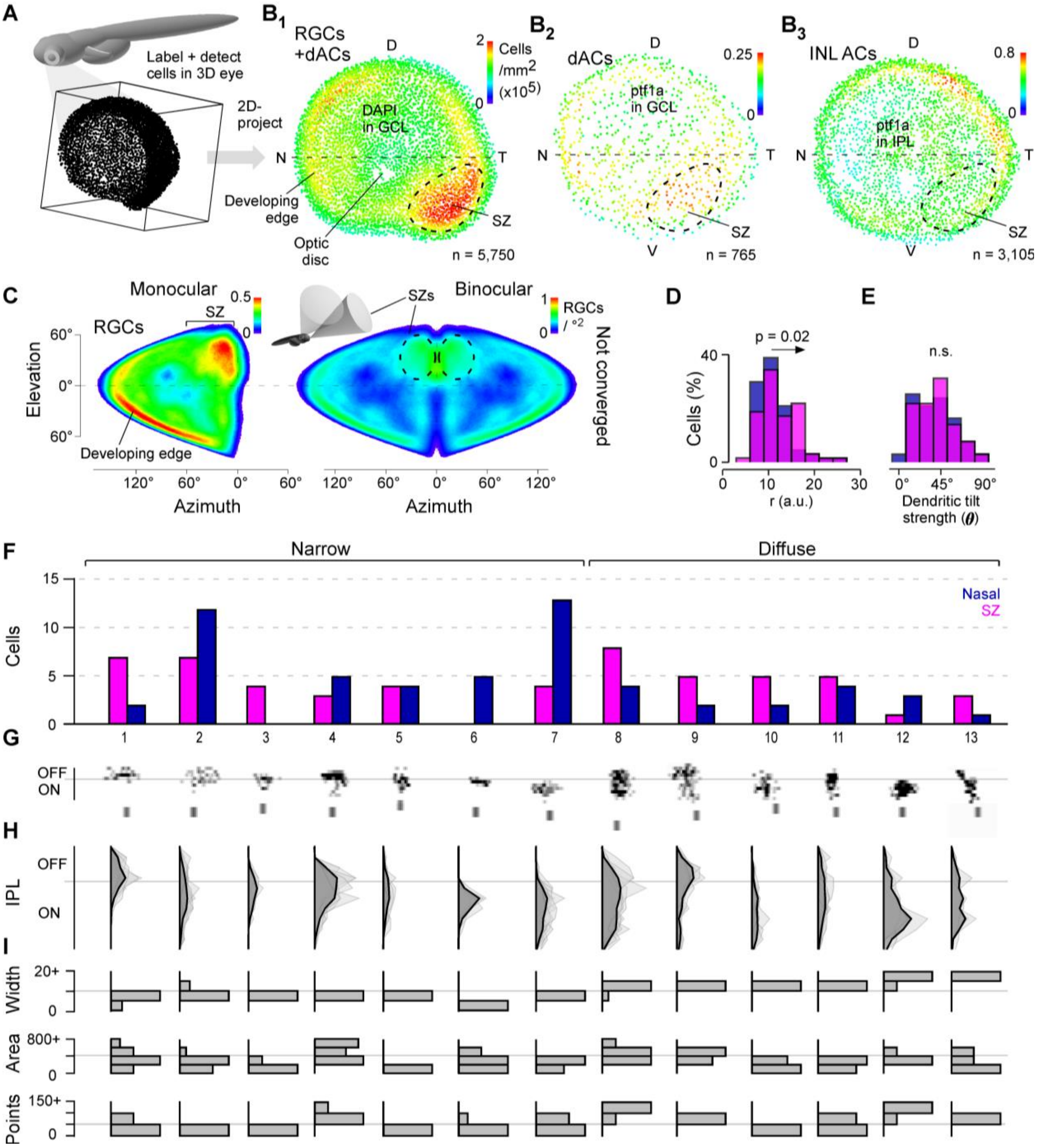

Figure S6. Further analysis on RGC anatomy. Related to Figure 7. A, Schematic of larval zebrafish and enlarged 3D representation of GCL nuclei in the eye. **B1-3**, 2D projections of detected soma positions across the eye of all GCL cells based on a DAPI stain which includes all RGCs and all dACs (1) and selective isolation of amacrine cells in the GCL (dACs , 2) and INL (ACs, 3) based on ptf1a labelling. N = 1 eye. **C**, Projections of RGC densities into monocular (left) and binocular (right) visual space during ‘rest (eyes not converged). **D,E**, Summary histograms of the distributions of r and φ (cf. Figure 7G) for quantifying dendritic tilt in photo labelled RGCs, pink: SZ, purple: Nasal. The distribution of r was weakly but significantly right shifted in SZ RGCs relative to nasal RGCs (D), while the corresponding distributions of φ were non-statistically distinct (E). Both: Two-sample Kolmogorov-Smirnov test. **F-I**, summarises an asymmetric distribution of anatomical RGC types across the eye. Photoconverted and processed RGCs from both nasal and SZ (cf. Figure 7D-M) were jointly clustered based on morphological criteria (Methods). **F**, Number of RGCs for SZ (pink/ left) and nasal retina (blue/right) allocated to each of n = 13 clusters. **G**, Individual RGC morphologies representative for each cluster. Note that each morphology’s depth profile (y) is stretched five-fold relative to its lateral

spread (x) to highlight stratification differences between clusters. **H**, Mean (dark) and individual depth profiles (light) and **I**, distribution of widths, dendritic field area and number of puncta for each cluster. Clusters were divided into narrow (left) and diffusely stratified (right) based on their mean widths (I, top, cf. labels in F).
